# Soft Bioelectronic Interfaces for Continuous Peripheral Neural Signal Recording and Robust Cross‐Subject Decoding

**DOI:** 10.1002/advs.202414732

**Published:** 2025-05-28

**Authors:** Liangpeng Chen, Yang Liu, Yewei Huang, Ziyang Li, Chao Zhang, Jun Tang, Xueyuan Ling, Bowen Cao, Baowang Li, Yuan Zhang, Wenjianlong Zhou, Qin Xu, Shunchang Ma, Xiudong Guan, Dan Xiao, Jingyao Geng, Yutong Zhao, Guolin Li, Yi‐xuan Wang, Wang Jia, Yuanwen Jiang, Milin Zhang, Deling Li

**Affiliations:** ^1^ Department of Neurosurgery, Beijing Tiantan Hospital, National Center for Neurological Disorders Capital Medical University Beijing 100070 China; ^2^ Department of Electronic Engineering Tsinghua University Beijing 100084 China; ^3^ State Key Laboratory of Space Network and Communications Tsinghua University Beijing 100084 China; ^4^ Department of Materials Science and Engineering University of Pennsylvania Philadelphia 19104 USA; ^5^ Beijing Neurosurgical Institute Capital Medical University Beijing 100070 China; ^6^ China National Clinical Research Center for Neurological Diseases (NCRC‐ND) Beijing 100070 China; ^7^ Tianjin Key Laboratory of Molecular Optoelectronic Sciences, Department of Chemistry, School of Science Tianjin University Tianjin 300072 China; ^8^ Institute for Precision Medicine Tsinghua University Beijing 100084 China

**Keywords:** cross‐subject decoding, neural network decoding, PEDOT:PSS, peripheral neural signal recording, soft bioelectronic interface

## Abstract

Accurate decoding of peripheral nerve signals is essential for advancing neuroscience research, developing therapeutics for neurological disorders, and creating reliable human–machine interfaces. However, the poor mechanical compliance of conventional metal electrodes and limited generalization of existing decoding models have significantly hindered progress in understanding peripheral nerve function. This study introduces low‐impedance, soft‐conducting polymer electrodes capable of forming stable, intimate contacts with peripheral nerve tissues, allowing for continuous and reliable recording of neural activity in awake animals. Using this unique dataset of neurophysiological signals, a neural network model that integrates both handcrafted and deep learning‐derived features, while incorporating parameter‐sharing and adaptation training strategies, is developed. This approach significantly improves the generalizability of the decoding model across subjects, reducing the reliance on extensive training data. The findings not only deepen the understanding of peripheral nerve function but also open avenues for developing advanced interventions to augment and restore neurological functions.

## Graphical abstract

A soft poly (3,4‐ethylenedioxythiophene):poly (styrenesulfonate)‐based electrode enables continuous, high‐quality recording of peripheral nerve activity. A neural network model integrating handcrafted and convolutional neural network‐based features decodes whisker movements with strong generalization, offering insights into peripheral nerve function and potential applications for therapeutic intervention.

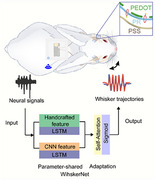

## 1 Introduction

Accurate decoding of neural signals is essential for elucidating fundamental physiological mechanisms,^[^ ^1^ ^]^ developing treatments for neurological disorders,^[^ ^2^ ^]^ and creating human–machine interfaces for prosthetic devices^[^ ^3^ ^]^ and communication applications.^[^ ^4^ ^]^ While substantial progress has been made in neurotechnology for the central nervous system, where simultaneous recording from hundreds of neurons in the brain has become routine practice, current technologies for interfacing with the peripheral nervous system remain relatively underdeveloped.

Challenges associated with studies of peripheral nerves mainly stem from their unique physiological characteristics. Unlike the brain, which is protected by a rigid skull, peripheral nerves experience significant mechanical deformation and displacement during movement.^[^ ^5^ ^]^ As a result, neural‐interfacing devices must exhibit tissue‐level mechanical compliance to maintain stable contact with minimal invasiveness. In addition, peripheral nerve signals, compared to those from the central nervous system, are characterized by sparse firing patterns and weak axonal propagation, often lacking large, synchronized events. This results in significantly smaller signal amplitudes.^[^ ^5^, ^6^ ^]^ To reliably capture such low‐amplitude signals, electrode materials must have ultralow contact impedance to ensure high sensitivity and precision in neural recordings.

Among current peripheral nerve interfaces technologies, intraneural electrodes, which penetrate the epineurium to make direct contact with individual fascicles or fibers, are commonly adopted.^[^ ^7^ ^]^ Although limited success has been achieved in capturing motor signals for prosthetic control, the long‐term use of such devices is severely constrained by irreversible damage to neural tissues, including physical trauma to the nerves, disruption of interfascicular blood flow, and the formation of glial scars.^[^ ^8^ ^]^ In contrast, extraneural devices such as cuff electrodes offer the advantages of minimal invasiveness and potentially greater longevity. However, the protective perineurium and epineurium layers attenuate the signal amplitudes at the electrode interface, limiting signal quality. In addition, conventional cuff electrodes, due to their bulk and rigidity, struggle to maintain stable contact in the dynamic environment of the body.^[^ ^9^ ^]^ Consequently, current extraneural devices typically provide only intermittent recordings in quasi‐static conditions, making it difficult to achieve continuous, high‐fidelity neural recordings during dynamic physiological states.

In addition to the reliable acquisition of raw neural activity, accurate across‐subject decoding represents another significant challenge, primarily due to the substantial variability in behaviorally relevant signal characteristics. This variability arises from differences in axon recruitment patterns, fascicular structures, vascularization, as well as variations in electrode implantation locations.^[^ ^6^, ^10^ ^]^ Several strategies have been developed to address these complex and often unpredictable factors. One common approach is feature transformation, where the system adapts to varying signal characteristics across subjects. However, this method is often time‐consuming and labor‐intensive, as it requires extensive feature extraction across subjects and frequent electrode repositioning to match expected implantation sites, resulting in a repetitive and burdensome training process.^[^ ^11^ ^]^ Another strategy is parameter fine‐tuning, which requires making minimal adjustments to the model's architecture and training process. While less labor‐intensive, this method risks overfitting the model to subject‐specific features, thereby compromising its generalizability.^[^ ^11^, ^12^ ^]^ Ideally, a robust decoding model for cross‐subject applications should effectively balance the preservation of prior knowledge while adapting to the unique characteristics of new subjects.

**Figure 1:**
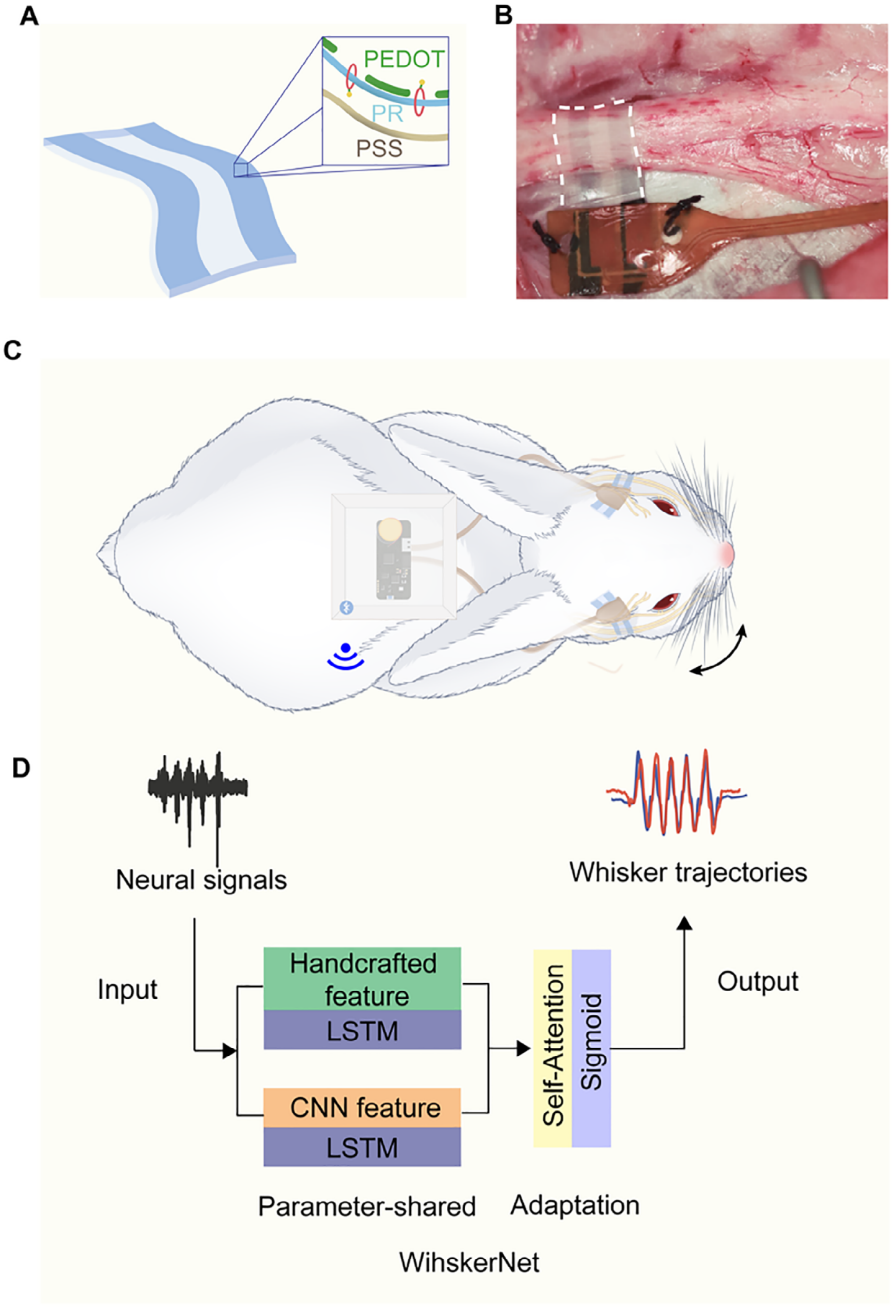
Schematic diagram of peripheral nerve signal recording and decoding strategy. A) Schematic diagram illustrating the rail conductive polymers electrode array and the major components and construction. PR, polytrioxane. B) Photograph illustrating the PEDOT:PSS electrodes wrapped around the facial nerve for signal recording, achieving stable enclosure. C) Schematic diagram illustrating the peripheral nerve signal recording in fully awake animals. D) Schematic diagram illustrating the neural signal decoding model.

In this study, we utilized low‐impedance, soft, conducting polymer electrodes to form intimate, stable interfaces with peripheral nerves (**Figure** **1**A,B), enabling reliable and continuous monitoring of neural activity in awake animals (Figure 1C). Leveraging the unique dataset collected from these recordings, we developed a neural network model capable of accurately predicting movement trajectories across multiple subjects (Figure 1D). This integrative approach addresses the critical challenges in peripheral nerve signal acquisition, while establishing a robust framework for neural decoding. Ultimately, this method holds significant potential for advancing neural interface technologies, offering applications in the augmentation, supplementation, and restoration of peripheral nerve function.

## 2 Results and Discussion

To demonstrate the efficacy of our strategy for neural signal recording and decoding, we focused on the facial nerve, which plays a central role in expressing emotions and interpersonal communication. Facial paralysis, resulting from impairment of the facial nerve, affects approximately 120 individuals per 100 000 each year and severely diminish quality of life.^[^ ^13^ ^]^ However, current treatments, such as physical therapy, nerve and muscle transfer have limited effectiveness in fully restoring facial nerve function.^[^ ^14^ ^]^ In response to this unmet clinical need, we developed a soft conducting polymer electrode based on poly(3,4‐ethylenedioxythiophene):poly(styrenesulfonate) (PEDOT:PSS), which was implanted around the facial nerve to enable continuous neural signal recording in awake animals model. Futhermore, we implemented a robust neural decoding model capable of generalizing across subjects. This integrated appraoch provides new insights into facial nerve function and offers a potential novel therapeutic approach for facial paralysis.

### 2.1 Characterization of the PEDOT:PSS Conductive Polymer Electrodes

Due to poor geometric and biomechanical compliance with the nerve, existing extraneural electrodes struggle to maintain stable contact in the dynamic environment of the body. As a result, most of these electrodes primarily offer intermittent nerve recordings, providing only brief snapshots of neural activity at isolated time points, often under anesthesia (Table S1, Supporting Information). Such approaches may inadequately capture the real‐time dynamics required to establish a robust relationship between neural activity and functional task representation.^[^ ^5^, ^15^ ^]^ To address this gap, we developed a low impedance PEDOT:PSS electrode array to continuously record peripheral nerve signals in fully awake animals, to better reflecting physiological conditions.

**Figure 2:**
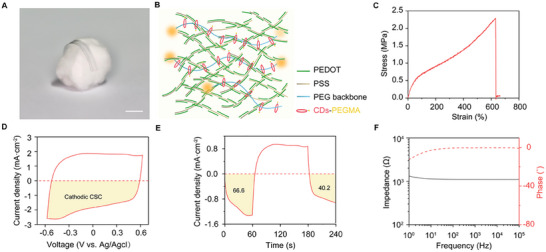
Overview of PEDOT:PSS conductive polymer electrode arrays. A) Photograph of the PEDOT:PSS conductive polymer electrode arrays on a cotton ball, highlighting its flexible and lightweight construction. B) Schematic diagram illustrating the interaction between PEDOT:PSS and topological supramolecular network via polytrioxane (PR) cross‐linking. PEG, polyethylene glycol; CDs, cyclodextrins; PEGMA, PEG methacrylate. C) Strain–stress curves demonstrating the mechanical properties of the PEDOT:PSS conductive polymers. D) Cyclic voltammetry curve of the PEDOT:PSS conductive polymers with the highlighted region (yellow) showing the integral of the cathodic current used for cathodic CSC estimation. E) Current density plotted as a function of time, specifying the value obtained from the integral of the highlighted area (yellow). F) Electrochemical impedance spectroscopy of the PEDOT:PSS conductive polymers.

The rail conductive polymers electrode array based on PEDOT:PSS was used on the facial nerve interface (**Figure** **2**A). The details of array synthesis and characterization were described in our previous studies.^[^ ^16^ ^]^ In brief, this stretchable conducting polymer electrode was based on PEDOT:PSS, integrated with a topological supramolecular network via polytrioxane (PR) cross‐linking. The network consists of a polyethylene glycol (PEG) backbone and sliding cyclodextrins (CDs) functionalized with PEG methacrylate (PEGMA) side chains to induce stretchability and high conductivity (Figure 2B).

The PEDOT:PSS electrode exhibits remarkable mechanical ductility, with a stretchability exceeding 600%, thereby ensuring reliable performance under significant deformation (Figure 2C). In addition, the mechanical modulus of the electrode is ≈360 kPa, which aligns well with the mechanical properties of peripheral nerve interfaces.

To assess electrode performance in bioelectronic applications, the charge storage capacity (CSC) was estimated by measuring the time integral of the current over the voltage range during cyclic voltammetry (CV), which provided a measure of how much charge the electrode can store. In the field of neural interfaces, it is commonly accepted to use the cathodic CSC as a benchmark for comparing different electrodes.^[^ ^17^ ^]^ In our study, PEDOT:PSS conductive polymers possessed a cathodic CSC of 106.8 mC cm^−2^, as calculated from the time integral of the enclosed area highlighted in yellow shade (Figure 2D,E). This value was much higher than that of the conventional platinum electrode with an estimated CSC of 0.5 mC cm^−2^.^[^ ^18^ ^]^ Furthermore, electrochemical impedance spectroscopy (EIS) results demonstrated that the impedance of PEDOT:PSS remained around 1.1 kΩ across a wide frequency range, from 1 to 10^5^ Hz (Figure 2F). This was advantageous for achieving high‐fidelity recordings and required lower input for effective stimulation.

### 2.2 Stable Recording of Behaviorally Linked Nerve Activity in Awake Animals

**Figure 3:**
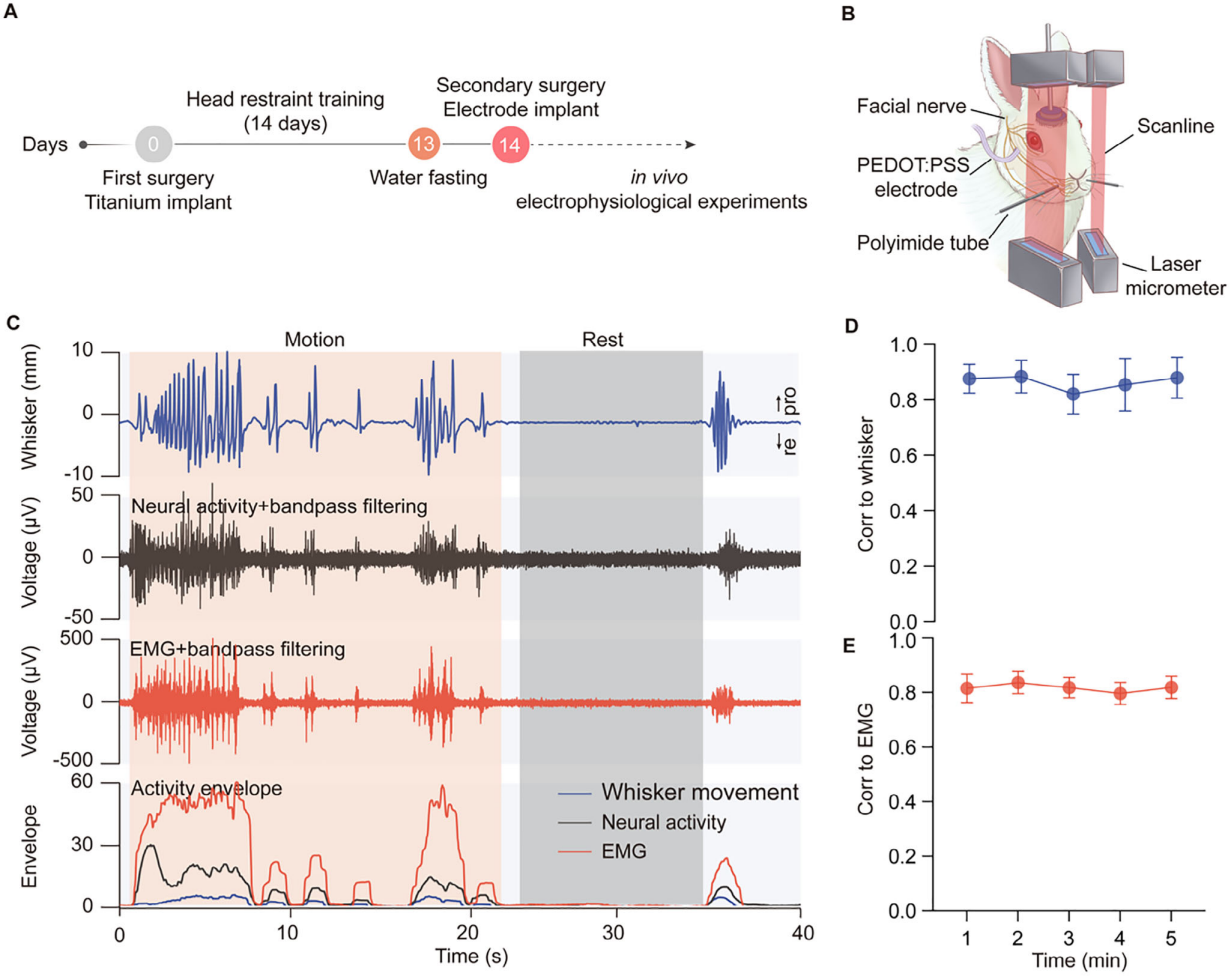
Experimental setup and synchronized signal recordings during whisker movement monitoring in awake rabbits. A) Experimental timeline depicting key procedures. Adult male New Zealand rabbits underwent initial surgery (day 0) for titanium screw implantation to enable head fixation. After a 14‐d head restraint training period, a secondary surgery was performed for electrode implantation. This was followed by in vivo signal recording experiments. B) Schematic of the in vivo signal recording experiments. The rail PEDOT:PSS conductive polymers electrode arrays were implanted around the zygomatic and buccal branches of the facial nerve for neural signal recording. Laser micrometers were positioned on either side of the rabbit's face to monitor whisker movements. The restraint apparatus included a head and bodies fixation system to minimize interference during whisker trajectory tracking. C) Representative traces of simultaneously recorded signals. Top row: whisker motion trajectories, with protraction (pro) and retraction (re) directions marked. Second row: bandpass‐filtered neural activity of the facial nerve branches. Third row: bandpass‐filtered EMG signals of the whisker pad muscle. Bottom row: signal envelopes of whisker movement, neural activity, and EMG signals. The red‐shaded areas indicated the whisker movement period, while the grey‐shaded area indicated the rest period. D,E) Pearson's correlation between neural signal envelopes and whisker movement envelopes (D), and neural signal envelopes and EMG envelopes (E) over a 5‐min recording period. D) The correlation between the neural signal envelope and the whisker movement envelope showed a strong positive relationship across the recording period (1 min, 0.88 ± 0.05; 2 min, 0.88 ± 0.06; 3 min, 0.82 ± 0.07; 4 min, 0.85 ± 0.09; 5 min, 0.88 ± 0.07). Points represented the mean correlation calculated per second (*n* = 6). E) The correlation between the neural signal envelope and the whisker pad EMG envelope also exhibited a significant positive relationship (1 min, 0.81 ± 0.05; 2 min, 0.84 ± 0.04; 3 min, 0.82 ± 0.04; 4 min, 0.80 ± 0.04; 5 min, 0.82 ± 0.04). Points represented the mean correlation calculated per second (*n* = 6).

To rigorously evaluate the performance of the PEDOT:PSS electrodes for signal acquisition in awake animals, we conducted simultaneous recordings of facial nerve activity, whisker movement trajectories, and EMG signals from the whisker pad muscle, which is innervated by the facial nerve (**Figure** **3**A,B). The nerve activity recording demonstrated consistent, high‐amplitude multi‐unit activity that was closely associated with whisker movements, in marked contrast to the periods of whisker resting state (Figure 3C, top and second row). The root‐mean‐square (RMS) amplitude during whisker movement was 13.31 ± 1.70 µV, indicating robust neural activity. In comparison, during whisker resting states, the background noise exhibited an average RMS of 5.58 ± 0.30 µV, which was low and stable across trials, confirming the high quality of the recordings. Furthermore, clear synchronizations in both the temporal and amplitude domains between the nerve activity and EMG signals of the whisker pad muscles were observed (Figure 3C, second and third row). The nerve activity envelope showed a strong correlation with both the whisker movement and EMG envelopes, with all exhibiting similar time‐varying trajectories (Figure 3C, bottom row).

For a quantitative assessment of recording stability, we computed trial‐by‐trial Pearson's correlations between the nerve activity envelope and both the whisker movement trajectory and whisker pad EMG signal envelopes over 1‐min intervals. The mean correlation was 0.86 ± 0.07 for the whisker movement trajectory envelopes (Figure 3D) and 0.82 ± 0.04 for the EMG signal envelopes (Figure 3E). Notably, no significant variation in these correlations was detected from the initial minute through subsequent time points (all *P* > 0.05, Figure S1A,B, Supporting Information), indicating the stability of signal relationships over the recording duration. In addition, we evaluated the efficacy of the electrode array in sustaining stable neurophysiological recordings in fully moving animals. The findings indicated that the PEDOT:PSS interfaces consistently maintained stable and high‐quality neural activity recordings in freely moving rabbits (Figure S1C and Movie S1, Supporting Information). The low modulus and impedance of the PEDOT:PSS electrode array enabled close conformity to the nerve,^[^ ^16^, ^19^ ^]^ thereby maintaining robust tissue‐electrode contact and accommodating nerve dynamics in awake animals, which ensured sustained and reliable signal acquisition.^[^ ^5^, ^9^ ^]^

### 2.3 Neuronal Origin of Recorded Signals

Despite the excellent electrical and mechanical properties of PEDOT:PSS electrodes, which facilitate stable recordings from peripheral nerves in awake animals, recording conditions in such complex environments can be confounded by contamination from EMG signals or motion artifacts.^[^ ^5^, ^10^ ^]^ Neural signals recorded using extra‐neural electrodes typically exhibit amplitudes in the range of tens of microvolts, significantly smaller than EMG signals, which are often orders of magnitude larger.^[^ ^5^, ^6^, ^9^ ^]^

**Figure 4:**
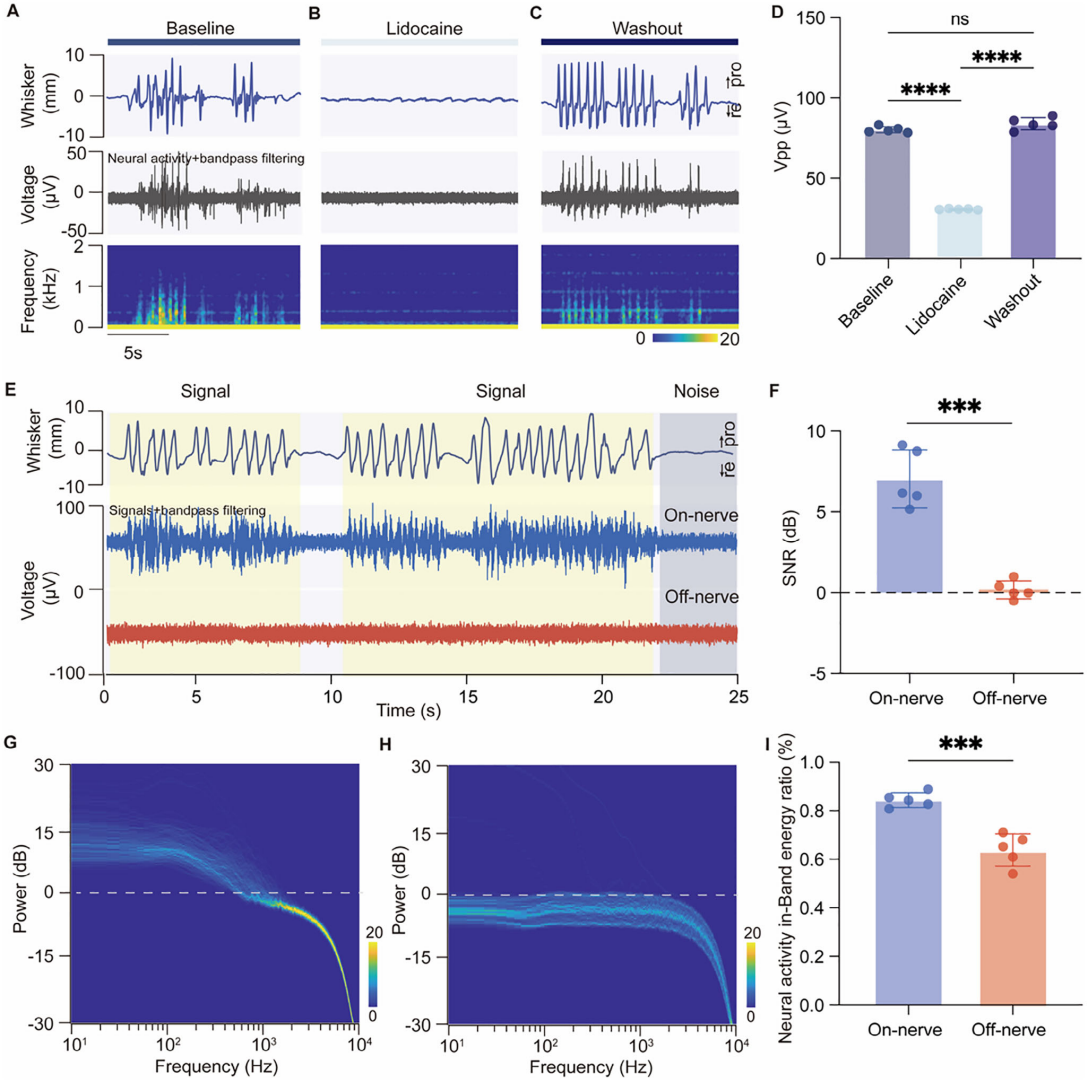
Validation of the neural origin of facial nerve recordings. A–C) Representative examples of neural signal responses to lidocaine blockade experiments. A) Baseline whisker motion (top), corresponding neural signals (middle), and spectrogram (bottom). B) Lidocaine blockade eliminates neural activity and reduces whisker motion. C) Recovery of neural activity and whisker motion post‐lidocaine washout. D) Quantification of peak‐to‐peak voltage (Vpp) across conditions. A significant reduction in Vpp was observed following lidocaine treatment (baseline, 80.28 ± 1.94 µV vs lidocaine, 30.51 ± 0.32 µV; *P* < 0.0001, Tukey's test), with no significant difference between baseline and washout (84.00 ± 3.80 µV; *P* = 0.072, Tukey's test). Points represented the mean Vpp of the signal of 500 whisker motion periods in each rabbit (*n* = 5). E) Representative examples of on‐nerve and off‐nerve recording. Yellow‐shaded regions denoted whisker‐related signal recording; gray‐shaded regions indicated noise. F) Trial‐by‐trial signal‐to‐noise ratio (SNR) for on‐nerve and off‐nerve recordings. On‐nerve signals showed significantly higher SNR compared to off‐nerve signals (on‐nerve, 7.05 ± 1.78 dB vs off‐nerve, 0.18 ± 0.56 dB; *P* < 0.001, paired *t*‐test). Points represented the mean SNR of the signal of 500 whisker motion periods in each rabbit (*n* = 5). G,H) Power spectra for on‐nerve and off‐nerve signals during whisker motion. I) In‐band energy ratio (sub‐1500 Hz) of on‐nerve and off‐nerve recordings. On‐nerve signals exhibited a higher in‐band energy ratio (on‐nerve, 0.85 ± 0.03 vs off‐nerve, 0.64 ± 0.08; *P* < 0.001, paired *t*‐test). Points represented the mean in‐band energy ratio of the neurophysiological signal in each rabbit (*n* = 5). ***P < 0.001, ****P < 0.0001, ns, not significant.

To confirm the neuronal origin of recorded signals from the facial nerve, we performed lidocaine blockade experiments.^[^ ^9c^ ^]^ Lidocaine (2.0% in saline) was applied to the main trunk of facial nerve to block nerve conduction. At baseline, transient fluctuations in the value of peak‐to‐peak (Vpp) amplitude of neural activity associated with whisker movement were observed (baseline, 80.28 ± 1.94 µV, **Figure** **4**A, top and middle row). Spectral analysis revealed significant power in the 100–1500 Hz range during whisker movements, with clear frequency ridges (Figure 4A, bottom row), along with similar spectral patterns in the lower frequency band (10–100 Hz; Figure S2A, Supporting Information). After lidocaine blockade, both whisker movements and the amplitude variation in neural activity were abolished, leaving only continuous low‐frequency physiological noise (Figure 4B). Spectrogram analysis further confirmed the absence of signal power in the 10–1500 Hz range, relative to baseline (Figure S2B, Supporting Information). The Vpp was significantly reduced (lidocaine, 30.51 ± 0.32 µV; *P* < 0.001). Following washout, neural activity amplitudes returned to baseline levels (Figure 4C; Figure S2C, Supporting Information), with no significant difference from baseline values (washout, 84.00 ± 3.80 µV; *P* = 0.072; Figure 4D). These results provided confirmation of the neuronal origin of the recorded signals, with the characteristic frequency band of whisker‐related neural activity observed to be below 1500 Hz.

To evaluate the contribution of non‐neuronal signals to the overall recorded activity, we conducted simultaneous recordings using PEDOT:PSS based electrodes positioned both directly on the facial nerve branches (on‐nerve) and in the connective tissue adjacent to the nerve (off‐nerve). The off‐nerve electrode was implanted ≈5 mm from the connective tissue adjacent to the neural tissue, while both the off‐nerve and on‐nerve electrodes were aligned in parallel and distal to the whisker pad muscles, ensuring minimal interference from muscle activity. The on‐nerve recordings exhibited robust signals with substantial amplitude fluctuations, characteristic of neural activity, whereas the off‐nerve recordings displayed poorly defined signals with minimal amplitude variation (Figure 4E).

To quantitatively assess the relative contribution of neuronal and non‐neuronal components, we calculated the signal‐to‐noise ratio (SNR) of whisker‐related signals on a trial‐by‐trial basis, using each whisker‐free recording as the in vivo noise floor. The SNR analysis revealed a significant difference in SNR between on‐nerve and off‐nerve signals across animals (on‐nerve: 7.05 ± 1.78 dB; off‐nerve: 0.18 ± 0.56 dB; *P* < 0.001, Figure 4F), underscoring the predominant contribution of neuronal signals in the on‐nerve recordings.

To further delineate the components of recorded signals, we performed a comprehensive frequency domain analysis comparing on‐nerve and off‐nerve recordings during whisker motion and rest states. On‐nerve recordings exhibited a broader distribution and higher energy density of energy in the sub‐1500 Hz range during whisker motion, compared to a more concentrated distribution observed during whisker rest (Figure 4G; Figure S3A, Supporting Information). In contrast, off‐nerve signals displayed a noise‐like and uniform energy distribution across all frequency bands, regardless of whisker movement (Figure 4H; Figure S3B, Supporting Information). Most importantly, during whisker motion, the on‐nerve recordings showed a significantly broader energy distribution and higher energy density in the sub‐1500 Hz range compared to off‐nerve recordings.

A detailed trial‐by‐trial statistical analysis of the neural activity in‐band energy ratio confirmed these findings. Specifically, the proportion of sub‐1500 Hz energy relative to the total signal energy during motion states was significantly higher in on‐nerve recordings compared to rest states (on‐nerve motion: 0.85 ± 0.03 vs on‐nerve rest: 0.74 ± 0.02, *P* = 0.01; Figure S3C, Supporting Information). Moreover, this proportion was also significantly elevated in comparison to off‐nerve recordings during whisker motion (off‐nerve motion: 0.63 ± 0.07, *P* < 0.001; Figure 4I). Notably, no significant difference was observed between motion and rest states in off‐nerve recordings (off‐nerve rest: 0.63 ± 0.07, *P* = 0.999; Figure S3C, Supporting Information), suggesting that off‐nerve recordings did not effectively capture whisker‐related neural activity. The distinct difference in both the frequency distribution and energy intensity between on‐nerve and off‐nerve recordings, combined with the absence of significant SNR in off‐nerve recordings during whisker movement, strongly suggested that the recorded signals from the on‐nerve electrode predominantly originated from neuronal activity, with minimal contamination from EMG signals or motion artifacts.

These findings provided compelling evidence supporting the neuronal origin of signals recorded using PEDOT:PSS electrode arrays, with negligible interference from non‐neuronal components such as EMG signals and motion artifacts contamination. The stability and specificity of the recorded signals demonstrated the efficacy of PEDOT:PSS‐based electrode arrays for consistent and reliable neural signal acquisition in awake, freely behaving animals.

### 2.4 Decoding Model Architecture and Training Strategy

Decoding of peripheral nerve signals remains a significant challenge in neuroscience due to several key limitations, including low SNR and the contamination of nerve signals with EMG activity and motion artifacts, as peripheral nerves are anatomically close to muscle tissue.^[^ ^5^, ^10^ ^]^ In addition, significant intersubject variability in the neural signal, arising from differences in axon recruitment patterns, fascicular structures, vascularization, as well as variations in electrode implantation locations, introduces further complexity, making it difficult to achieve robust and accurate generalization of decoding models across individuals.^[^ ^6^, ^10^, ^15^ ^]^ To overcome these obstacles, we utilized the above unique neurophysiological dataset of the facial nerve to develop an advanced neural decoding framework, which we refer to as “WhiskerNet”. This framework integrates both parameter‐sharing and adaptation strategies to enhance cross‐subject decoding accuracy.

**Figure 5:**
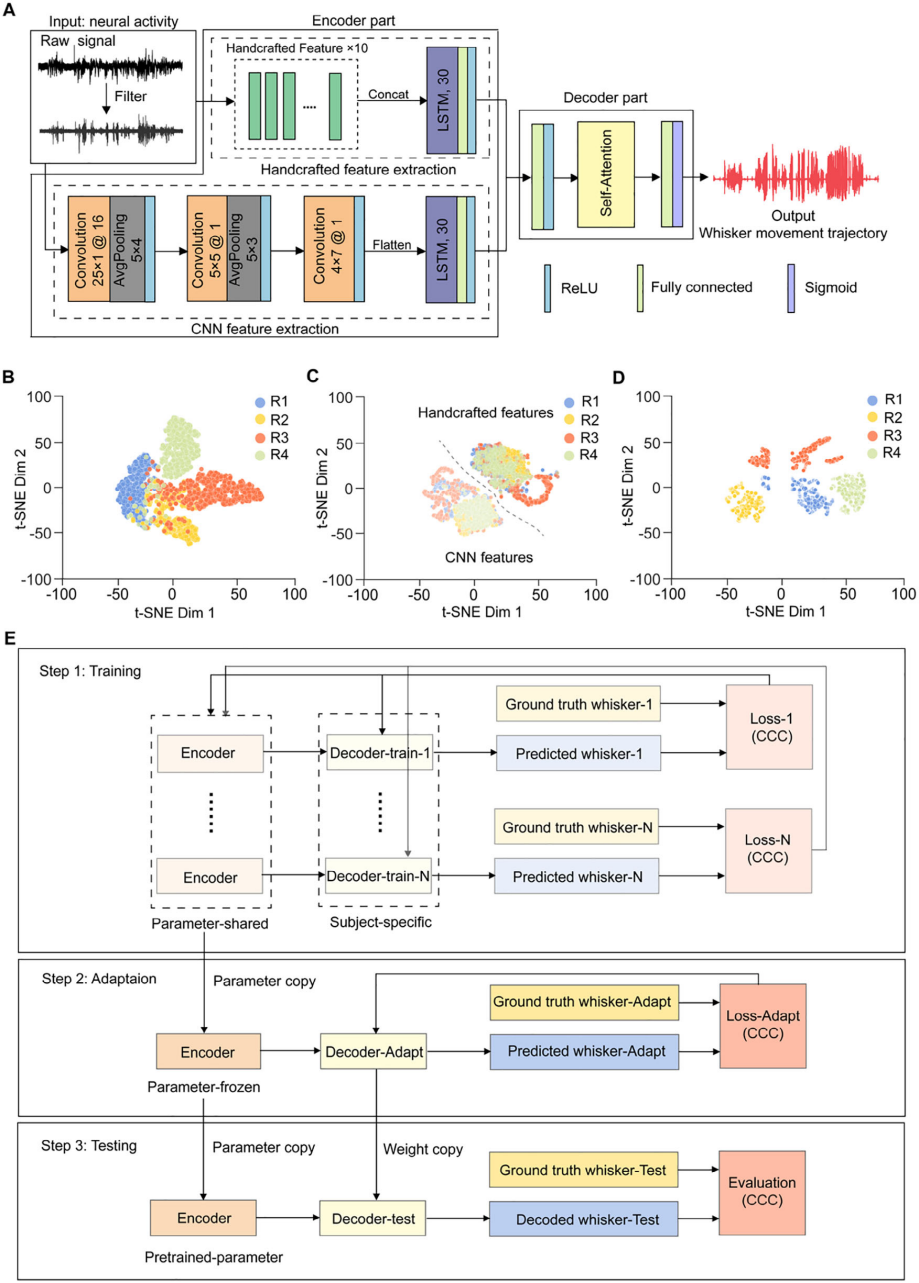
WhiskerNet architecture and decoding model training strategy. A) Schematic of the WhiskerNet model. The encoder part extracts feature through two parallel pathways: handcrafted feature extraction (green) and CNN feature extraction (orange). The decoder part uses a self‐attention mechanism to predict whisker movement trajectories. B–D) t‐SNE visualizations of neural activity features across four subjects (R1–R4, *n* = 4). B) Distribution of raw signal features including mean absolute value, simple square integral, average amplitude change, and standard deviation. C) Outputs from the encoder part, showing distinct clustering between handcrafted (dark) and CNN (light) features, separated by a gray dashed line. D) Outputs before the final fully connected decoder layer, revealing subject‐specific distributions. E) Training strategy of WhiskerNet. Step 1. Training: the encoder was shared across all subjects, with subject‐specific decoders trained individually. During training, the encoder's parameters were updated. Step 2. Adaptation: a reinitialized decoder adapted to the new test subject using a subset of their data, while the encoder's parameters remained frozen. Step 3. Testing: the WhiskerNet was tested using a shared encoder for all subjects and a personalized decoder for each test subject.

WhiskerNet integrates handcrafted and convolutional neural networks (CNN) for feature extraction with long short‐term memory (LSTM) units to transform neural signals into latent vectors, which are then used for predicting whisker motions at specific time intervals (**Figure** **5**A). The handcrafted feature extraction combines expert‐driven manual features with LSTM units that capture temporal dynamics in neural activity. Concurrently, the CNN, augmented with their own LSTM units, autonomously learn multiscale, hierarchical features from the raw neural signals.^[^ ^20^ ^]^ To optimize the feature extraction process, we implemented a feature fusion strategy where the CNN learns spatial patterns from the neural signals, which are then temporally refined using LSTM units. This approach enables the model to adaptively focus on varying time scales, ranging from rapid fluctuations to sustained neural patterns, thus providing a more accurate feature extraction of whisker movements during both high‐frequency bursts and prolonged motor activities. The extracted features from both the handcrafted and CNN pathways are integrated through fully connected layers in the decoder.^[^ ^21^ ^]^ A self‐attention mechanism models the temporal dependencies between neural signals and motor behavior, enhancing the model's ability to decode whisker movements across subjects with high accuracy.^[^ ^22^ ^]^ This architecture ensures robust adaptation to inter‐subject variability, addressing the significant challenge posed by variability in neural signals and supporting reliable neural decoding in diverse individuals.

To further clarify the functional stages within the WhiskerNet architecture, we conducted signal visualization at various points in the network across four subjects. Initially, prior to network input, we extracted trial‐by‐trial features from raw signals, including mean absolute value (MAV), simple square integral (SSI), average amplitude change (AAC), and standard deviation (STD). These features exhibited substantial intersubject variability, highlighting significant differences in neural signal characteristics in diverse individuals (Figure 5B). Following the handcrafted feature extraction and CNN‐based processing, the outputs for all subjects coalesced into two distinct clusters within the feature space, delineated by a clear boundary. This clustering underscored the effectiveness of feature fusion in ensuring comprehensive feature extraction and dimensionality reduction (Figure 5C). Finally, just before the last fully connected layer, the decoder outputs revealed distinct, subject‐specific distributions, demonstrating the model's capability for individualized signal characteristics integration (Figure 5D). These visualizations illustrated the network's ability to integrate personalized neural signal features while maintaining inter‐subject distinctions, thus enhancing the accuracy of subject‐specific neural decoding.

To improve the generalization capacity of WhiskerNet across subjects, we incorporated parameter‐sharing and adaptation strategies into the model training process (Figure 5E). During the training phase, the encoder's parameters were updated collectively across all training subjects, while each subject was assigned a distinct, personalized decoder, whose parameters were updated exclusively with subject‐specific data. This design ensured that the shared encoder captured generalizable neural signal features, while the decoder retained subject‐specific variations. In the adaptation phase, the frozen encoder, shared from the training subjects, was applied to new subjects. For each new subject, a reinitialized decoder was trained using a subset of their data, allowing the model to adapt to individual signal characteristics. Thus, in the final testing configuration, WhiskerNet employed a pre‐trained encoder—generalizing feature extraction across subjects—combined with a newly trained, subject‐specific decoder. This hybrid approach ensured the stability and universality of the feature extraction process while enabling personalized decoding, significantly enhancing the model's ability to generalize across diverse subjects.

### 2.5 Cross‐Subject Decoding Performance of the WhiskerNet

Most peripheral nerve decoding studies focus on within‐subject training, with little emphasis on cross‐subject generalization. As summarized in Table S2 (Supporting Information), prior approaches largely relied on handcrafted features and classical machine learning models (e.g., SVM, Kalman filters) or CNN‐based architectures, which lack adaptability across individuals and require extensive calibration. These limitations restrict their broader applicability, particularly for real‐world neural interface applications. In contrast, WhiskerNet introduces a parameter‐sharing and adaptation strategies to enhance cross‐subject robustness. By integrating self‐attention mechanisms into the adaptive prediction head, WhiskerNet not only improves decoding accuracy but also reduces data requirements for new subjects, facilitating wider translational applications.

**Figure 6:**
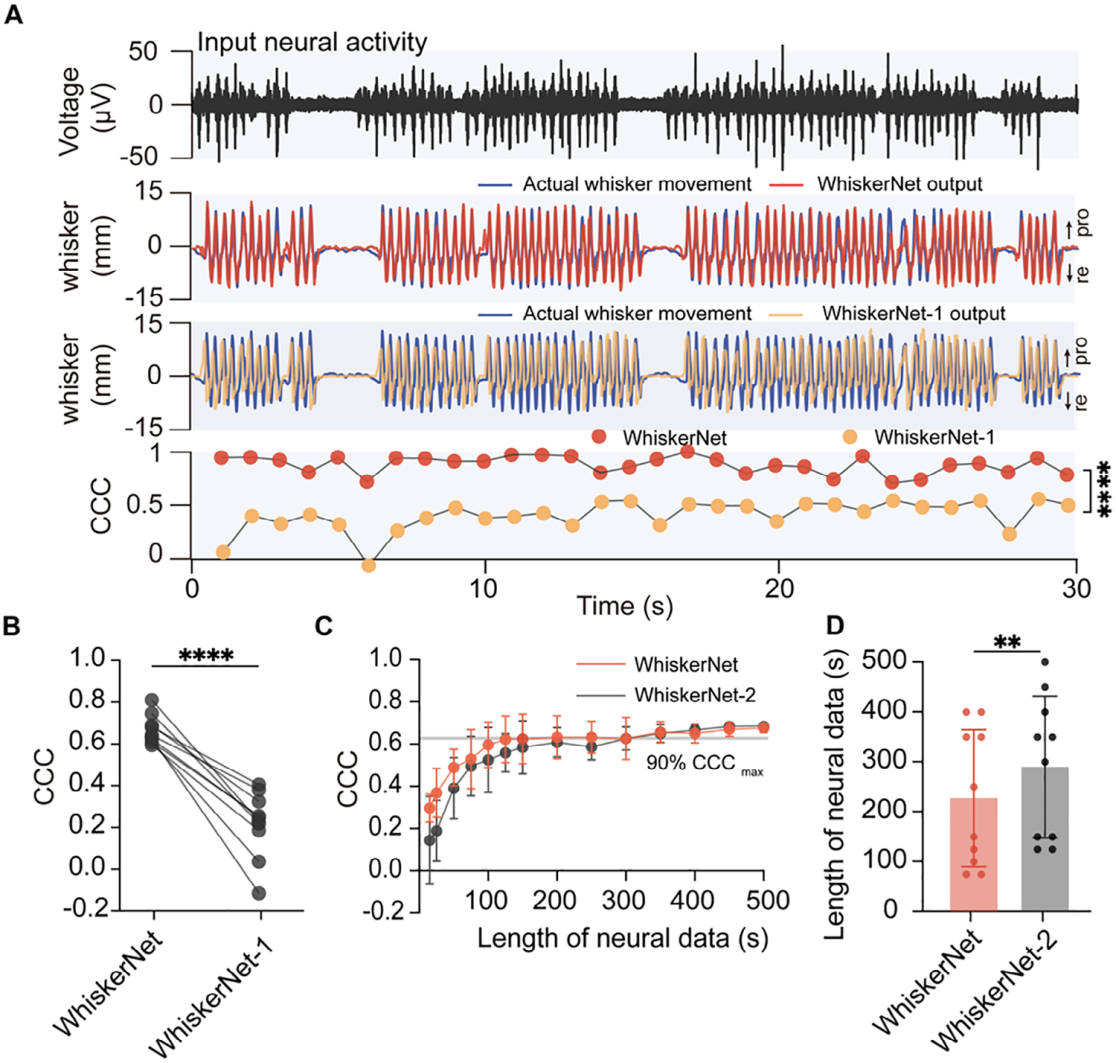
Cross‐subject decoding performance of WhiskerNet. A) Examples of decoding performance for WhiskerNet and WhiskerNet‐1 (control model with no adaptation). Top: bandpass‐filtered neural signals serve as the input to the decoding network model. Second: actual whisker movement trajectories (blue) and WhiskerNet output (red), with protraction (pro) and retraction (re) directions indicated. Third: actual whisker movement trajectories (blue) and WhiskerNet‐1 output (yellow). Bottom: trial‐by‐trial concordance correlation coefficients (CCC) between the actual whisker movement trajectories and decoded whisker trajectories. WhiskerNet showed superior performance (0.85 [IQR, 0.76–0.89]) compared to WhiskerNet‐1 (0.53 [IQR, 0.43–0.59]; *P* < 0.0001, repeated‐measures ANOVA). Points represented the mean CCC calculated per second between the output of WhiskerNet (red) and WhiskerNet‐1 (yellow), and the actual whisker movement trajectories in the test subject. B) Decoding performance comparison between WhiskerNet and WhiskerNet‐1. The inclusion of adaptive decoding in WhiskerNet significantly improves decoding accuracy (CCC: 0.68 ± 0.07 vs 0.23 ± 0.17; *P* < 0.001, paired *t*‐test). Points represented the CCC between the output of WhiskerNet and WhiskerNet‐1, and the actual whisker movement trajectories in each test subject (*n* = 10). C) Learning curve of CCC performance over increasing neural data for WhiskerNet and WhiskerNet‐2 (control model with no parameter sharing). WhiskerNet requires less neural data to converge to 90% of its maximum performance compared with WhiskerNet‐2. Points represented the CCC between the output of WhiskerNet (red) and WhiskerNet‐2 (black), and the actual whisker movement trajectories at different neural data lengths in test subject. D) Comparison of the data lengths required for model training between WhiskerNet and WhiskerNet‐2 for the new subjects. A significant difference was observed for the data length needed to achieve 90% of the maximum CCC between WhiskerNet and the WhiskerNet‐2 (WhiskerNet, 200.0 ± 119.6 s vs WhiskerNet‐2, 280.0 ± 141.3 s; *P* = 0.007, paired *t*‐test). Points represented the length of neural data needed to achieve 90% of the maximum CCC (*n* = 10). **P < 0.01, ****P < 0.0001.

To evaluate its cross‐subject performance, we developed a control model, WhiskerNet‐1, in which both encoder and decoder parameters were shared across all subjects without adaptation. Filtered neural signals were then fed into both WhiskerNet and WhiskerNet‐1 (**Figure** **6**A, top row), generating corresponding outputs. Compared to the actual whisker movement trajectories, WhiskerNet's output exhibited strong alignment (Figure 6A, second row), whereas WhiskerNet‐1′s output displayed clear delays and inconsistencies in amplitude (Figure 6A, third row). Notably, WhiskerNet performed optimally during active whisker movements, although its accuracy diminished slightly during rest states (Figure S4, Supporting Information). The concordance correlation coefficient (CCC) analysis further highlighted WhiskerNet's superior decoding capability relative to WhiskerNet‐1 for the new test subject (WhiskerNet: 0.85 [IQR,0.76 – 0.89] vs WhiskerNet‐1: 0.53 [IQR,0.43 – 0.59], *P* < 0.0001; Figure 6A, bottom row).

To account for potential biases introduced by varying datasets, a tenfold cross‐validation procedure was implemented for both models, alternating between training and testing subjects. These results demonstrated a significant advantage for WhiskerNet over WhiskerNet‐1 (CCC: WhiskerNet: 0.68 ± 0.07 vs WhiskerNet‐1: 0.23 ± 0.16, *P* < 0.001; Figure 6B). This performance gain can be attributed to the subject‐specific decoder in WhiskerNet, which, following the adaptation process, facilitated individualized integration of extracted features, and effectively compensated for signal‐motor latencies across subjects, ensuring accurate and reliable decoding in a cross‐subject context.

In addition, to investigate whether shared parameters can effectively transfer the useful information and enhance cross‐subject decoding performance, we trained another control model, WhiskerNet‐2, in which the parameters of both the encoder and decoder components were fully individualized for the test subjects without utilizing any prior parameters from the training sets. The results demonstrated that, with minimal training data, WhiskerNet significantly outperformed WhiskerNet‐2. As the size of the training dataset increased, both models showed gradual improvements, eventually plateauing at similar performance levels. However, throughout this process, WhiskerNet maintained a consistently higher performance than WhiskerNet‐2 (Figure 6C).

To quantitatively evaluate the reduction in training data required by the parameter‐sharing model, we established a benchmark at 90% of the maximum performance. Across ten subjects, WhiskerNet required, on average, 28.6% less training data to reach this benchmark compared to WhiskerNet‐2 (WhiskerNet: 200.00 ± 119.60 vs WhiskerNet‐2: 280.00 ± 141.30, *P* = 0.007; Figure 6D). These findings suggest that the feature extraction parameters learned from the training sets are generalizable to new subjects, requiring only minimal updates to the decoder part, thus enabling rapid convergence with significantly reduced training data and effort for new subjects.^[^ ^23^ ^]^

### 2.6 Conclusion and Outlook

In this study, we introduce a novel paradigm for stable, continuous recording and accurate decoding of neural signals from peripheral nerves. Unlike existing extraneural nerve interfaces, our soft, low‐impedance PEDOT:PSS‐based conducting polymer electrode enabled reliable, continuous recording of peripheral neural activity in awake animals. Leveraging this unique neurophysiological dataset, we developed a neural network model that integrates handcrafted temporal features with CNN‐based features, utilizing parameter‐sharing and adaptation strategies to achieve high accuracy decoding of whisker movement across subjects. The robustness of this model, characterized by its strong generalization capability and reduced data requirements for new subjects, suggests its potential for broader application. This approach holds promise for decoding a wide range of motor neural signals, such as the governing blink, ocular movement, and limb motion. Importantly, this work not only advances the understanding of peripheral neural signal processing but also lays the groundwork for novel therapeutic interventions aimed at enhancing or restoring peripheral nerve function.

Although the system successfully integrates key features for both signal recording and decoding, further optimizations are essential to enhance its generalization capacity. Notably, during stationary whisker states, the model generates residual noise instead of a zero output, highlighting its vulnerability to minor input perturbations. This suggests that the current neural network framework lacks sufficient robustness against small‐scale variances in the input.^[^ ^24^ ^]^ Moreover, the inherent time‐varying and noisy nature of neural signals detracts from the regression accuracy over time, particularly in sequential data processing.^[^ ^25^ ^]^ To address these limitations, future developments should focus on incorporating data augmentation strategies and training with adversarial samples to improve the model's resilience to corrupted inputs and ensure more reliable performance in variable conditions.

In addition, long‐term in vivo implantation studies in animal models are crucial for assessing the durability and consistency of signal recording and decoding performance under chronic conditions.^[^ ^5^, ^26^ ^]^ Future directions should focus on the development of chronically stable, adhesive, soft, and stretchable electrode arrays, integrated with robust, self‐adaptive decoding models. Such advancements are expected to not only deepen the understanding of underlying physiological mechanisms but also hold significant potential for clinical applications, including the restoration of peripheral nerve function.

## 3 Experimental Section

### Fabrication of PEDOT:PSS Conductive Polymer Electrode

PEDOT:PSS electrodes were prepared by mixing PEDOT:PSS aqueous dispersion (1.1 wt%, 1 mL, Orgacon ICP 1050, Agfa‐Gevaert N.V.) with a lab‐synthesized crosslinker polyrotaxane1 (55 mg), photoinitiator lithium phenyl‐2,4,6‐trimethyl‐ benzoylphosphinate (Sigma‐Aldrich, 1 mg), and fluorosurfactant (Zonyl FS‐30, Sigma‐Aldrich, 5 µL). The mixture was first filtered using nylon syringe filters (1 µm pore size, Whatman) and then spin‐coated onto the crosslinked SBS substrate at 500 r.p.m. for 15 s. The film was subsequently UV exposed through a photomask using a Spectrum 100 Precision UV Spot Curing System (American Ultraviolet) for 2 min, followed by development with water to remove the unexposed areas.

In the standard rail electrode configuration, each electrode features a width of 1 mm and is separated from its adjacent counterparts by a spacing of 1 mm.

### Mechanical Characterizations

Uniaxial tensile tests of the device, composed of patterned PEDOT:PSS electrode on an SBS substrate, were conducted using an Instron 5564 tabletop universal testing machine. Each sample was prepared with dimensions of 20 mm in length and 5 mm in width. Before testing, the entire sample was clamped in the fixture, leaving the original length at 10 mm for strain calculation. The thickness of the sample was measured to be 100 µm. During testing, uniaxial tensile test was performed at a strain rate of 50 mm min^−1^.

### Electrical and Electrochemical Characterizations

The following electrical and electrochemical measurements were acquired by a PalmSens4 electrochemical workstation.

EIS was performed by using PEDOT:PSS electrode as working electrode, Pt wire as counter electrode, silver/silver chloride (Ag/AgCl) as the reference electrode, and phosphate buffered saline (PBS, Thermo‐Fisher) as the electrolyte. The exposed area for PEDOT:PSS electrode was maintained with 1 mm × 1 mm. Impedance and phase angle as functions of frequency were acquired in response to sine wave signals amplitude of 10 mV. Cyclic voltammetry was performed by cyclically sweeping the voltage from −0.6 to +0.6 V with a scan rate of 20 mV s^−1^ for five cycles and recording the current signals. The CSC was calculated by the time integral of the cathodal currents in the potential range of water electrolysis window for PEDOT:PSS, then the charge was divided by the electrode surface area to provide the specific CSC in the unit of mC cm^−2^.

### Animal Subjects

The animal subjects for this study were adult male New Zealand rabbits. The care and experimental manipulation of the animals were performed following the guidelines of the Public Health Service Policy on Humane Care of Laboratory Animals and approved by the Beijing Neurosurgical Institution Laboratory Animal Welfare and Ethics Committee (Approval No. 202104010).

### Procedure of Titanium Screws Implantation and Head Fixation Training

Prior to in vivo electrophysiological experiments, each rabbit underwent surgery for the implantation of head fixation devices. The procedures were performed under isoflurane anesthesia (2%–4% in oxygen) and included perioperative analgesia with meloxicam (1%, Qilu Animal Health) and anti‐infection measures with enrofloxacin (5%, Baytril). A midline incision was made to expose the subperiosteal layer, and five titanium screws were implanted along the anterior and posterior edges of the bilateral frontal bone, as well as at the midpoint near the sagittal suture. Dental acrylic cement was applied to secure the screws, and the incision was closed.

Following surgery, rabbits underwent daily head restraint training, which continued until they acclimatized to two‐hour‐long experimental sessions. The head restraint apparatus included a pipe with a loop strap to restrict the rabbit's body, neck plates positioned at the front of the pipe to control head motion, and a perforated metal plate compatible with the titanium screws for additional head stabilization. This setup ensured the animals’ readiness for subsequent whisker trajectory motion tracking.

### Stretchable PEDOT:PSS Electrode Array for Signal Recording

For in vivo neurophysiological recordings, a rail PEDOT:PSS electrode array was fabricated with an electrode length of 4 cm and an interelectrode distance of 1 mm. A customized flexible printed circuit (FPC; length: 20 cm; Shenzhen V‐layer Printed Circuit Technology Co.) was fabricated to connect the PEDOT:PSS electrodes via a two‐channel gold finger interface and to link to the wireless neural signal acquisition device using a four‐channel gold finger interface. The reference and ground electrodes were positioned 5 cm distal to the two‐channel gold finger. The PEDOT:PSS electrodes were attached to the FPC's two‐channel gold finger contacts using carbon tape (Figure S5A, Supporting Information). To maintain structural stability and ensure the integrity of the connections, the interface assembly was encapsulated with silicone elastomer (Kwik‐Sil, WPI).

### Device Parameter of Wireless Neural Signal Acquisition Device

A customized miniaturized wireless neural signal acquisition device was developed to collect and wirelessly transmit neural signal data (NeuraMatrix.llc; Figure S5B, Supporting Information). The data acquisition front‐end comprised an analog‐front‐end (AFE) chip and a Bluetooth Low Energy (BLE) data transmission module integrated on an nRF5340 microcontroller unit (MCU) (Figure S5C, Supporting Information). The AFE included a low‐noise chopper amplifier (LNA) with a DC‐servo loop (DSL), a programmable gain amplifier (PGA), a 13‐bit successive approximation register (SAR) analog‐to‐digital converter (ADC), a Cascaded Integrator‐Comb (CIC) low‐pass filter (LPF), a power management unit (PMU), a lead‐off detector, and a digital controller. The PMU comprised low dropout regulators (LDO), a charge pump (CP), and a bandgap voltage reference circuit (BGR). Communication between the AFE chip and MCU was facilitated via the Quad Serial Peripheral Interface (QSPI) protocol (Figure S4D, Supporting Information). The BLE module enabled wireless transmission of quantized data at a maximum rate of 32 kHz, with a BLE dongle and host computer receiving and storing the transmitted signals.

The AFE chip was fabricated using 180 nm complementary metal‐oxide‐semiconductor (CMOS) technology and supported a variable sampling frequency ranging from 250 Hz to 32 kHz. The integrated input‐referred noise of the AFE was 0.79 µV over a frequency range of 1 Hz to 1 kHz, with a spurious‐free dynamic range of 76 dB using a 93 Hz, 14 mV peak‐to‐peak sinusoidal input signal. The wireless neural signal acquisition device consumed 30.13 mW of power and was powered by a 100 mAh lithium coin cell, allowing for continuous operation for at least 10 hours.

### Procedure of Rail PEDOT:PSS Electrode Array Implantation

Rabbits underwent a 12‐h water fasting period before the electrode implantation procedure. The protocol for general anesthesia and perioperative management was consistent with that used in the initial surgery. First, the masseter muscle was separated from the zygomatic arch, and the lower two‐thirds of the arch were excised to improve access for exposing the motor branch of the trigeminal nerve. Localized nerve blockade was achieved by administering 2.0% lidocaine in saline. Following this, A 2‐cm incision was made in the cheek to expose the zygomatic and buccal branches of the facial nerve, followed by the implantation of the rail PEDOT:PSS electrode array (Figure S6A–C, Supporting Information). The reference and ground electrodes were secured to the insides of the skin incision (Figure S6D, Supporting Information). The FPC was secured to the deep facial fascia over the masseter muscle using 5‐0 polypropylene sutures, with leads tunneled subcutaneously to exit the skin at the occipital region. Paired stainless steel microwires were inserted into the muscles of the whisker pad at 1‐cm intervals using a 23‐gauge hypodermic needle to record EMG signals.

### Electrophysiological Recording and Whisker Motion Tracking in Awake Animals

The electrophysiological signal recording and whisker motion trajectory tracking were conducted 6 h after electrode implantation, allowing sufficient time for the animals to recover from anesthesia and exhibit stable, continuous whisker movements under fixation.

### Electrophysiological Signal Recording

Neural activity and EMG signals were acquired using a wireless signal acquisition chip (NeuraMatrix.llc) in a common‐reference mode. During the signal analysis stage, neural activity was processed as common‐mode signals, while EMG signals were analyzed in a differential mode. Neural activity was sampled at 16 kHz and underwent bandpass filtering within the frequency range of 1–1500 Hz. For EMG signals, dual‐channel recordings were captured simultaneously. The bipolar EMG signal was reconstructed by computing the difference between the two recorded channels. EMG signals were sampled at 4 kHz and subjected to high‐pass filtering at 10 Hz.

### Whisker Motion Trajectory Tracking

Rabbits were positioned in a restraint apparatus to immobilize their heads and bodies, minimizing interference during whisker trajectory tracking. Two laser micrometers (IG‐208, Keyence Corp.) were placed in front of the rabbits’ faces to monitor the horizontal whisker motion trajectories. To enhance detectability, a single whisker (C‐1) was encased in a polyacrylamide tube with a diameter of 8 mm (Figure S7, Supporting Information). The micrometer was oriented parallel to the lateral surface at a 17° angle from the head midline and located 20 mm from the respective C1 origin. The spatial sensitivity of the micrometers was adjusted to exclusively detect the whisker within the tube while disregarding the other whiskers. Intermittent provision of small amounts of water proved effective in eliciting whisker movement. Whisker motion was sampled at a rate of 125 Hz with a high‐pass filter set at 1 Hz.

### Lidocaine Blockade Experiments

The extracranial segment of the main trunk of facial nerve was exposed at the junction between the mastoid process and the mandibular angle under general anesthesia with isoflurane. A subcutaneous catheter was implanted near the nerve for localized administration of the anesthetic. A total of 0.4 mL of 2% lidocaine solution (0.1 g:5 mL) was administered through the catheter to block the facial nerve. Following the lidocaine administration, the site was washed with sterile saline (3–5 times) to ensure complete removal of the anesthetic. Neural recordings were conducted 3–4 h post‐washout to ensure complete recovery of nerve conduction before data collection.

Neural signals were recorded before, during, and after the blockade, while EMG signals were simultaneously recorded to assess the effectiveness of the procedure.

### Electrophysiological Data Analysis

All offline analyses of electrophysiological data and whisker movement trajectories were performed using MATLAB (The MathWorks Inc. R2022b).

### Signal Alignment and Extraction of Whisker‐Related Neural Activity

Electrophysiological signals were aligned with whisker movement trajectories using cross‐correlation of signal envelopes to achieve temporal synchronization. Whisker‐related neural signals were extracted by segmenting neural activity corresponding to whisker movements. Motion periods were defined as intervals from the onset to the cessation of whisker movement, determined by detecting peaks in the whisker trajectory data. Neural signal segments associated with these peaks were extracted, segmented, and concatenated. Subsequently, the neural data aligned temporally with the whisker movement segments were merged, resulting in a dataset with neural activity specifically related to whisker movements.

### Electrophysiological Signals and Whisker Motion Trajectories Envelope Correlation

For each trial, Pearson's correlation between the envelope of electrophysiological signals and the envelope of whisker movement trajectories was calculated using a sliding window approach. The window width for whisker movement data was set to 200ms with a 100ms overlap, while for EMG data, the window width was 4 s with a 2 s overlap.

### Neural Activity Root Mean Square

The trial‐by‐trial RMS values of whisker‐related neural activity and noise were computed by calculating the RMS voltage for each whisker rest period within a sliding window (200 ms width, 100 ms overlap).

### Neural Activity Value of Peak‐to‐Peak

The trial‐by‐trial Vpp of whisker‐related nerve activity was computed as the difference between the maximum and minimum voltage recorded for each whisker movement period within a sliding window (width 200 ms, overlap 100 ms).

### Neural Activity Signal‐to‐Noise Ratio

The trial‐by‐trial SNR for on‐ and off‐nerve whisker‐related signals was determined using the formula 10 × log10 (RMS_S_ /RMS_N_) within a sliding window (width 200 ms, overlap, 100 ms), where RMS_S_ and RMS_N_ represented the root mean square of the signals with and without whisker movements, respectively. To establish the in vivo noise floor during non‐whisker recording, at least 10‐s segments were visually inspected to confirm the absence of whisker movement or other artifacts.

### Neural Activity Persistence Spectrum

The persistence spectrum was used to depict the distribution of neural activity frequencies and intensities over time. Signals were divided into 256 overlapping segments (50% overlap) and windowed with a Hamming function. The power spectrum of each segment was calculated using the fast Fourier transform (FFT), capturing the frequency intensity profile within each segment. The resulting frequency intensities were discretized into 256 evenly spaced levels, and these spectra were aggregated to form a comprehensive distribution of frequency and intensity.

### Neural Activity In‐Band Power Ratio

The in‐band power ratio was defined as the energy ratio within the 10–1500 Hz band to the total energy above 10 Hz, following a 50 Hz band‐stop filter application. Energy spectra were calculated using FFT within a sliding window (2 s width, 1 s overlap), allowing for trial‐by‐trial analysis of neural signal power dynamics.

### WhiskerNet Neural Network Architecture

The WhiskerNet architecture is designed with two primary components: an encoder and a decoder. The encoder includes two parallel feature extraction pathways: handcrafted feature extraction pathway and CNN‐based feature extraction pathway. The handcrafted pathway extracts predefined features from the signals, which are then processed through an LSTM network.^[^ ^26^, ^27^ ^]^ The CNN‐based pathway consists of multiple convolutional and pooling layers followed by an LSTM network to extract and refine features hierarchically. Outputs from both pathways are concatenated in the decoder, which integrates the extracted features and performs the remaining decoding operations (Table. S3, Supporting Information). All neural network components were implemented using PyTorch 2.0.1.

### Input and Output Processing

The WhiskerNet processed the input signal x[n], sampled at a rate of $f_{s}^{\mathrm{data}}$, and generated the output whisker movement y[n] at $f_{s}^{\mathrm{laser}}$. Input signals were segmented using a sliding window of length $N_{\mathrm{pad}}$ = 2000 samples with a stride of $f_{s}^{\mathrm{data}}/f_{s}^{\mathrm{laser}}$. The decoding function f computed the whisker position output as:

(1) $$y\left[n\right]=f\left(x\left[n\times f_{s}^{\mathrm{data}}/f_{s}^{\mathrm{laser}}-N_{pad}+1:n\times f_{s}^{\mathrm{data}}/f_{s}^{\mathrm{laser}}\right]\right)$$

The output y[n] represented the normalized whisker movements within the range of 0–1, with a baseline of 0.5 corresponding to the neutral position. This normalization reflects predicted whisker movements spanning a physical range of 0–28 mm as measured by the laser micrometer.

### Data Selection for Normalization

Prior to inputting the data into the network, both raw data and the extracted features were normalized for each individual to ensure that the input sample distribution had zero mean and unit variance. The mean and variance used for normalization were computed from the individual's own training set data. This approach ensures that the normalization process reflects the specific characteristics of each individual, minimizing any inter‐individual variability that may affect network performance.

For training, the normalization parameters were derived from 1000 s of data. For testing, a maximum of 500 s of adaptive data were used to calculate the normalization parameters. Importantly, the length of the test data was fixed at 500 s for all test individuals to maintain consistency across evaluation scenarios.

### Handcrafted Feature Extraction

Handcrafted features were extracted from the raw input of 2000 units using a sliding window with a length and stride of 125 units each (Table S4, Supporting Information).^[^ ^28^ ^]^ The extracted features were normalized using predefined mean and standard deviation values specific to each feature, derived from prior analysis of individual signal data. The normalized feature vectors were then processed through an LSTM network with a dimension of 160 × 30, followed by a fully connected layer sized 30 × 30.

### CNN Feature Extraction

The input data were first normalized using individualized parameters. The normalized data were then processed through a multilayer CNN beginning with one‐dimensional CNN (1D‐CNN) layer generating 16 channels. The output was reshaped into a two‐dimensional format and passed through a sequence of alternating average pooling layers and two‐dimensional CNN (2D‐CNN) layers. The architecture consisted of two average pooling layers and two single‐channel 2D‐CNN layers, facilitating comprehensive feature extraction from the normalized signal.

### Decoder Component

In the decoder, input data first passed through a fully connected layer, producing a feature vector $F_{t}$ at each time step t. A buffer maintained a sequence of previous outputs $\left\{F_{t-i}\in R^{60\;\times \;1}|i\;=\;1,2,\text{…},15\right\}$ for the preceding 15‐time steps. At each time step, the temporal self‐attention module accessed the current feature *F_t_* ​along with the buffered sequence and updated the buffer with $F_{t}$ for subsequent steps.^[^ ^22^ ^]^

The attention module transformed the 16 vectors into 30‐dimensional key, query, and value representations through separate fully connected layers with rectified linear unit (ReLU) activations, resulting in $\left\{K_{t-i}=Key\left(F_{t-i}\right)|i\;=\;0,1,2,\text{…},15\right\}$ and $\left\{V_{t-i}=Value\left(F_{t-i}\right)|i\;=\;0,1,2,\text{…},15\right\}$. Attention scores were calculated as:

(2) $$\begin{array}{c} S=\left[s_{0},s_{1},\text{…},s_{15}\right]=softmax\left(\left[K_{t}^{T}Q_{t}/4,K_{t-1}^{T}Q_{t-1}/4,\text{…},K_{t-15}^{T}Q_{t-15}/4\right]\right) \end{array}$$

The output of the attention module was computed as:

(3) $$O=\sum_{i=0}^{15}s_{i}V_{i}$$

The output from the attention layer was further condensed into a scalar value using a many‐to‐one fully connected layer, followed by a sigmoid activation function, yielding the final output within the range of 0–1.

### Parameter‐Sharing and Adaptation Strategies

To enhance generalization across subjects, parameter‐sharing and adaptation strategies were incorporated into WhiskerNet. The pretrained encoder, with shared parameters denoted as $W_{\mathrm{Enco}}$, was applied to new subjects. Adaptation involved assigning a subject‐specific decoder, with parameters $\;W_{Deco}^{i}$ for subject i.

(4) $$\left\{W_{Enco},W_{Deco}^{i}\right\}=\arg\;\min\;L\left(W_{Deco}^{i}\left(W_{Enco}\left(X^{i}\right)\right),Y^{i}\right)$$

During the training phase, for each training subject i within the training set, the parameters were updated by minimizing:

(5) $$W_{Enco},W_{Deco}^{i}=\arg\;\min\;L\left(W_{Enco},W_{Deco}^{i},X^{i},Y^{i}\right)$$

In the adaptation phase for a new testing subject, given samples $\left(X_{adapt}^{test},Y_{adapt}^{test}\right)$, the newly assigned decoder $W_{Deco}^{test}$ was trained as

(6) $$W_{Deco}^{test}=\arg\;\min\;L\left(W_{Enco},W_{Deco}^{test},X_{adapt}^{test},Y_{\mathrm{adapt}}^{test}\right)$$

During the testing phase for the new subject with input samples $X_{test}^{test}$, the predicted output ${\hat{Y}}_{test}^{test}$ was obtained using:

(7) $${\hat{Y}}_{\mathrm{test}}^{\mathrm{test}}=W_{\mathrm{Deco}}^{\mathrm{test}}\left(W_{\mathrm{Enco}}\left(X_{\mathrm{test}}^{\mathrm{test}}\right)\right)$$

### Loss Function

The criterion used for testing, training, and adaptation was the CCC,^[^ ^29^ ^]^ defined as:

(8) $$\rho_{c}\left(Y,\hat{Y}\right)=\frac{2\rho \sigma_{Y}\sigma_{\hat{Y}\;}}{\sigma_{Y}^{2}+\sigma_{\hat{Y}}^{2}+{\left(\;\mu_{Y}-\mu_{\hat{Y}}\right)}^{2\;}}$$

where ρ represents the Pearson correlation coefficient between the predicted sequence $\hat{Y}$ and the ground truth sequence Y. The terms $\sigma_{Y}$ and $\sigma_{\hat{Y}}$ are the standard deviations, while $\mu_{Y}$ and $\mu_{\hat{Y}}$ denoted the means of the respective sequences.

Given that CCC is a sequence‐based metric, the training and adaptation strategy employed a sequence‐to‐sequence approach. This method extended the input time‐series signals forward, allowing the model to generate outputs for multiple consecutive time points, thereby optimizing the performance on sequential data.

### Decoding Performance across Subjects

To evaluate the predictive capabilities of WhiskerNet across individuals, a tenfold cross‐validation approach was used. The dataset for each individual was split into training and testing sets, with the testing data held out during the training phase to avoid bias. In each fold, one individual was used as the test subject, and the model was trained on the remaining individuals. For each train‐test run, ten different random seeds (ranging from 1 to 10) were used for initialization to ensure robustness.

### Convergence Speed Testing

The model's convergence speed was assessed by applying a fivefold cross‐validation across different lengths of adaptive data. This procedure helped evaluate how quickly the model adapts to new individuals as the amount of training data varies. The fivefold cross‐validation ensures that results are averaged across multiple splits, providing a reliable estimate of convergence performance.

### Statistical Analysis

All statistical analyses were carried out using GraphPad (Prism 9.0). All replicate numbers, error bars, *P*‐values, and statistical tests are indicated in figure legends. The significance threshold for all two‐tailed tests was set at *P* < 0.05.

## Conflict of Interest

The authors declare no conflict of interest.

## Ethics Approval Statement

Beijing Neurosurgical Institution Laboratory Animal Welfare and Ethics Committee (Approval No. 202104010).

## Author Contributions

L.C. and Y.L. contributed equally to this work, listed in alphabetical order. Conceptualization: D.L., M.Z., Y.J., and W.J. Investigation: D.L., M.Z., Y.J., W.J., Y.W., and G.L. Methodology: L.C., Y.L.,Y.H., Z.L., C.Z., J.T., X.L., B.C., B.L., Y.Z., W.Z.,Q.X., S.M., X.G., D.X., J.G., Y.Z., G.L., Y.W., and Y.J. Software: L.C. and Y.L. Visualization: L.C., Y.L., and B.L. Writing‐original draft: L.C., Y.L., and Y.H. Writing‐review and editing: D.L., M.Z., Y.J., C.Z., Y.W., and G.L. Supervision: D.L., M.Z., Y.J., W.J., C.Z., and Y.W. Funding acquisition: D.L. and M.Z. Resources: D.L., M.Z., Y.J., and W.J.

## Supporting information



Supporting Information

Supplemental Movie 1

## Data Availability

The data that support the findings of this study are available from the corresponding author upon reasonable request.
