# Supplementary material for: Soft Bioelectronic Interfaces for Continuous Peripheral Neural Signal Recording and Robust Cross‐Subject Decoding
Source: Adv Sci (Weinh). 2025 May 28;12(33):e14732. doi: 10.1002/advs.202414732 (PMC12412577; doi:10.1002/advs.202414732)
Supplement: Supplementary file 1 — Supporting Information [file ADVS-12-e14732-s002.docx]

**
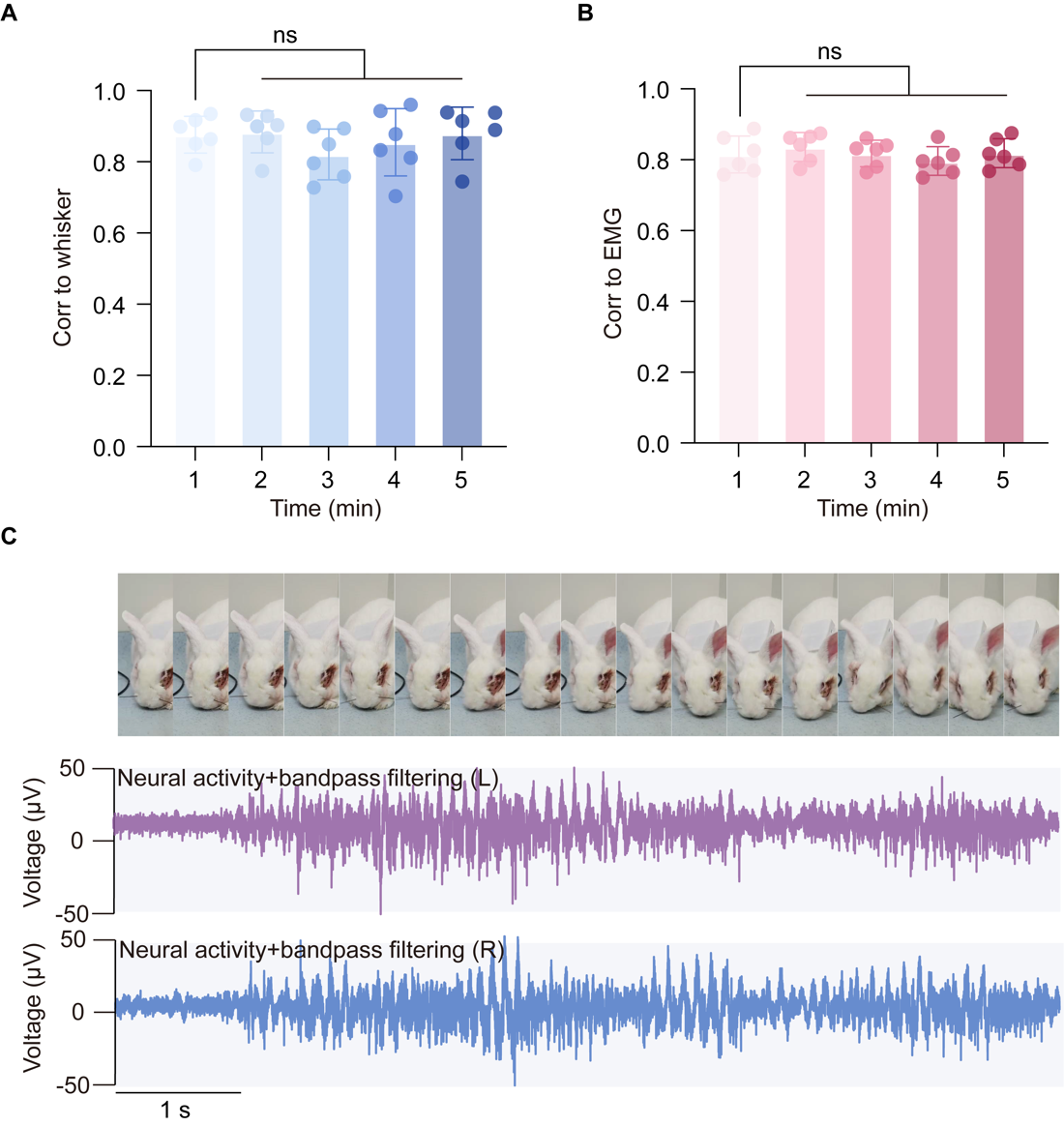
**

**Figure S1.**

Stable signal recording over time in fully awake animals. A) Pearson’s correlation between the neural signal envelope and whisker movement envelope over a 5-minute recording period (1 min, 0.88 ± 0.05; 2 min, 0.88 ± 0.06, *P* = 0.990; 3min, 0.82 ± 0.07, *P* = 0.359; 4min, 0.85 ± 0.09, *P* = 0.991; 5 min, 0.88 ± 0.07, *P* > 0.999, Tukey’s test). Points represented the mean correlation calculated per second (*n* = 6). B) Pearson’s correlation between the neural signal envelope and EMG signal envelope over a 5-minute recording period (1 min, 0.81 ± 0.05; 2 min, 0.84 ± 0.04, *P* = 0.619; 3min, 0.82 ± 0.04, *P* > 0.999; 4min, 0.80 ± 0.04, *P* = 0.444; 5 min, 0.82 ± 0.04, *P* > 0.999, Tukey’s test). Points represented the mean correlation calculated per second (*n* = 6). C) Representative examples of stable and continuous neural signal in free-moving rabbit. Top row: Photograph of the free-moving rabbit. Middle row: bandpass-filtered neural activity recorded of left facial nerve branches. Bottom row: bandpass-filtered neural activity recorded of right facial nerve branches. ns, not significant.

**
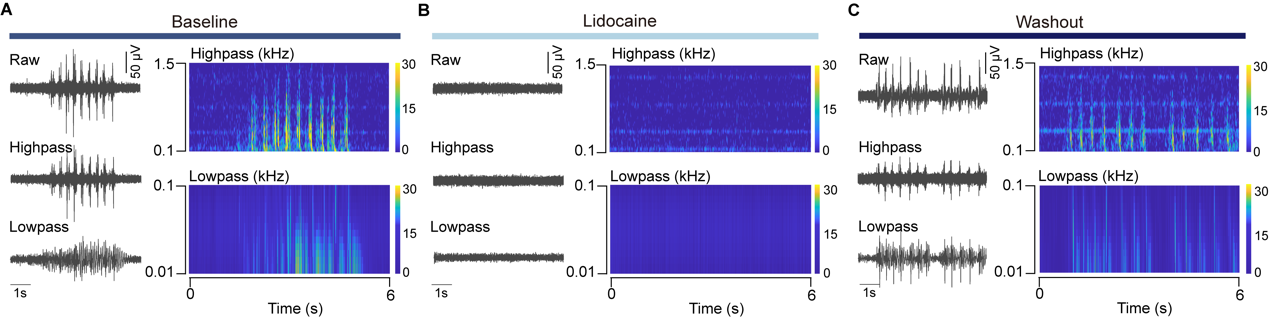
**

**Figure S2.**

Spectral analysis of neural activity during lidocaine blockade experiments. A) Baseline condition. Left: Neural signals after bandpass filtering (10 - 1500 Hz) (top), highpass filtering (> 100 Hz) (middle), and lowpass filtering ( <100 Hz) (bottom). Right: Spectral representations of highpass-filtered (top) and lowpass-filtered signals (bottom). B) Lidocaine administration. Neural signals after filtering (left) and their corresponding spectral analysis (right) as in A. Lidocaine significantly suppresses neural activity across all frequency bands. C) Washout period. Recovery of neural activity is observed with filtering and spectral analysis comparable to the baseline state.

**
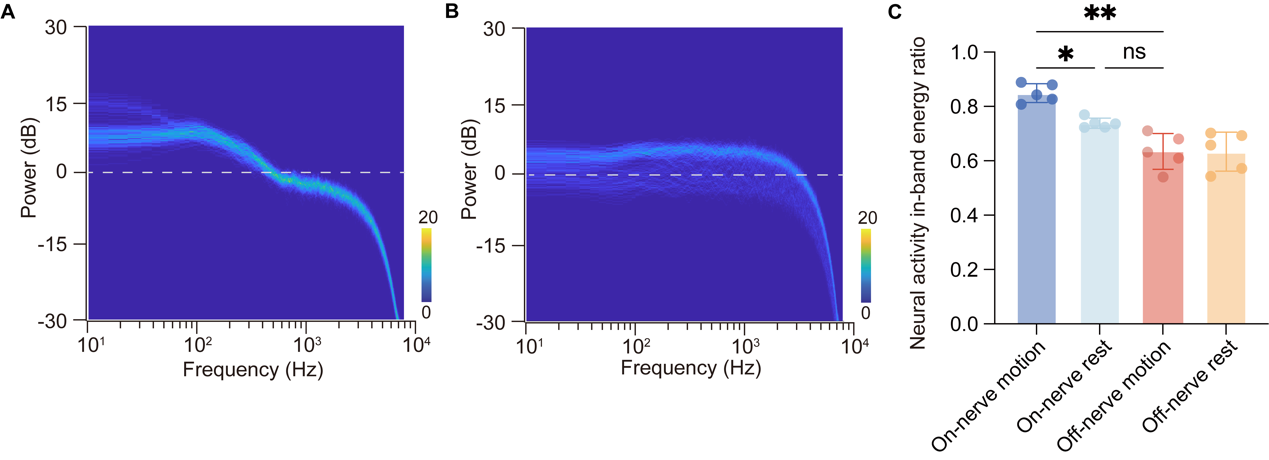
**

**Figure** **S3.**

Frequency characteristics of on-nerve and off-nerve signals during whisker motion and rest. A) Power spectral density of on-nerve neural recordings during rest, showing a more concentrated energy distribution predominantly below 1500 Hz. B) Power spectral density of off-nerve recordings during rest, displaying a uniform, noise-like energy distribution across all frequencies. C) In-band energy ratios (< 1500 Hz) for on-nerve and off-nerve recordings during whisker motion and rest. On-nerve recordings during whisker motion exhibited a significantly broader energy distribution and higher energy density in the sub-1500 Hz range compared to the more concentrated distribution during rest (*P* = 0.04, Tukey’s test). In contrast, off-nerve recordings showed no significant difference in energy distribution between whisker motion and rest conditions (*P* = 0.926, Tukey’s test). Points represented the mean in-band energy ratio of the neurophysiological signal in each rabbit (*n* = 5). *, *P* < 0.05, **, *P* < 0.001, ns, not significant.


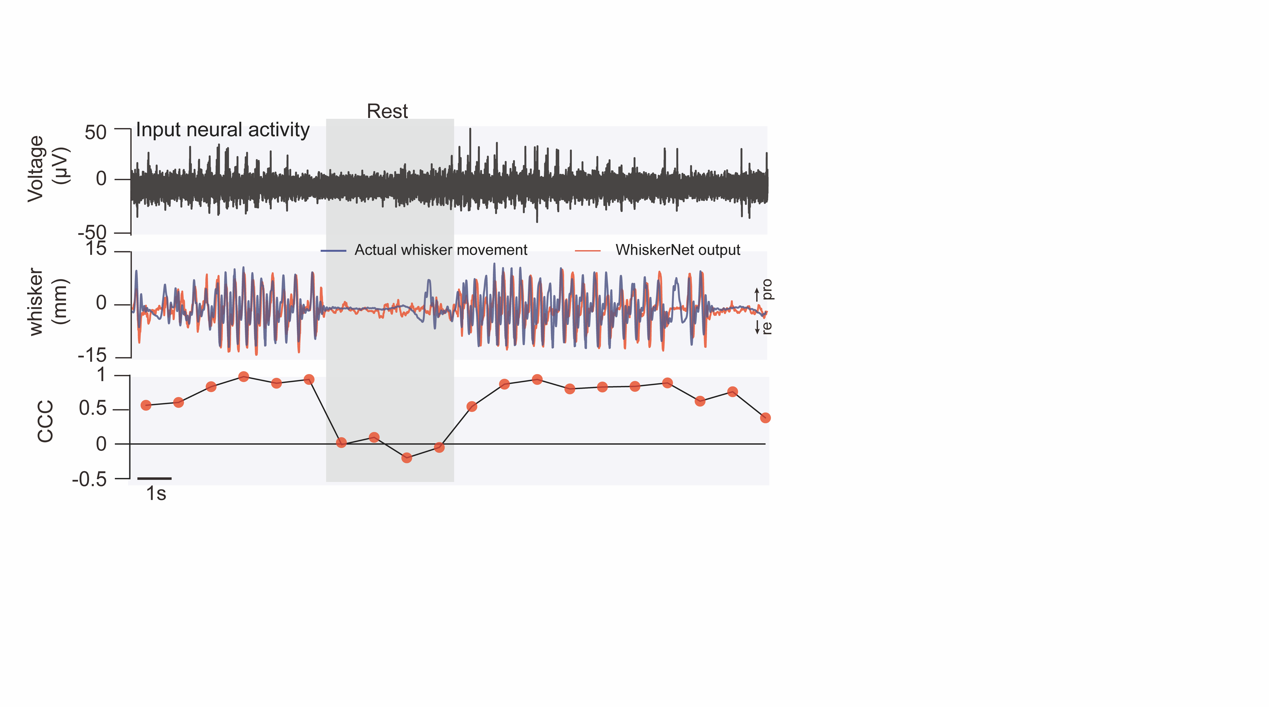


**Figure S4.**

Decoding performance of WhiskerNet during whisker rest periods. Top row: bandpass-filtered neural signals serve as the input to the decoding network model. Middle row: actual whisker movement trajectories (blue) and WhiskerNet output (red), with protraction (pro) and retraction (re) directions indicated. Bottom row: the trial-by-trial concordance correlation coefficient (CCC) between the actual whisker movement trajectories and WhiskerNet output. WhiskerNet maintained higher CCC values during whisker movement periods, with a noticeable drop during the rest period, indicating reduced decoding accuracy when whisker movement is minimal. Points represented the mean CCC calculated per second between the output of WhiskerNet and the actual whisker movement trajectories in the test subject. The grey-shaded region indicated the rest condition where whisker movement was minimal.

**
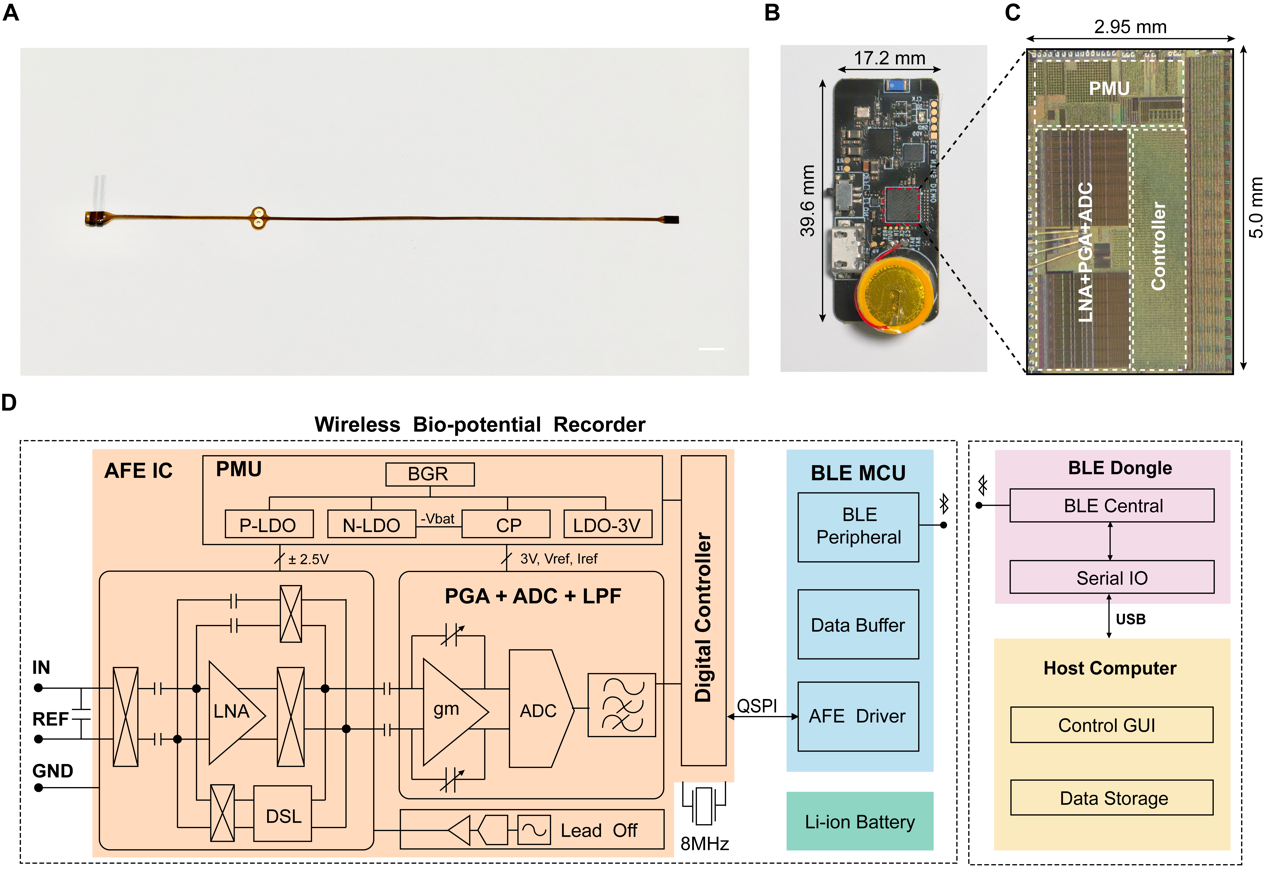
**

**Figure S5.**

Devices for the signal recording and transmission *in vivo* neurophysiology experiments. A) Photograph of the rail PEDOT:PSS electrode arrays and customized flexible printed circuits. B) Photograph of the miniaturized wireless neural signal acquisition device. C) Microphotography of the analog-front-end (AFE) chip. D) Schematic diagram of the wireless neural signal acquisition device. The data acquisition comprised an AFE chip, and a Bluetooth Low Energy (BLE) data transmission module integrated on a nRF5340 microcontroller unit (MCU).


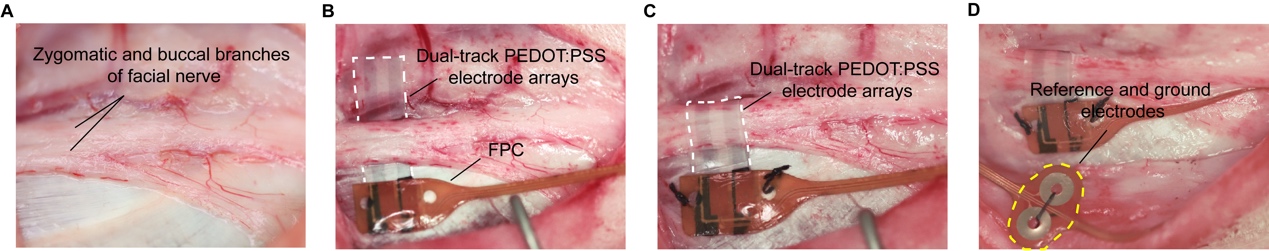


**Figure S6.**

Photograph of the PEDOT:PSS electrode array implantation process. A) Photograph of the zygomatic and buccal branches of the facial nerve. B-C) Photograph of the PEDOT:PSS electrodes wrapped around the facial nerve. D) Photograph of the reference and ground electrodes.

**
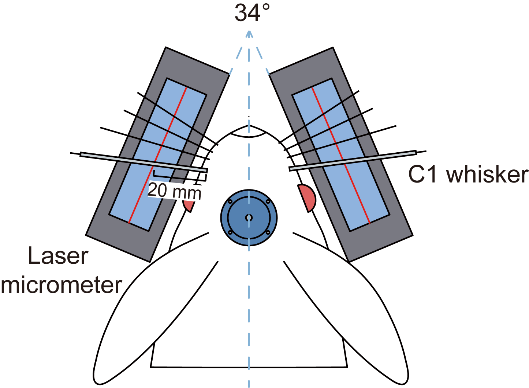
**

**Figure S7.**

Laser micrometer setup. Each micrometer was aligned parallel to the lateral surface, maintaining a 17° angle from the head midline. The origins of the C1 whiskers were centered on the laser micrometer scanlines, ensuring a consistent distance of 20 mm. Polyimide tubes were affixed to the C1 whisker, enabling the detection of the movement of the designated whisker.

| **Table S1. Comparison of extra-neural peripheral nerve interfaces.** | | | | | |
| --- | --- | --- | --- | --- | --- |
| Investigators | Interface type | Implant site | Animal state | Experiment | Results |
| Leob and Peck. et al. ^[1]^ | Split cuff: titanium and polyimide | Sciatic nerve; cat | Wakefulness | Recording, spontaneous and stimulation evoked | Vpp = 25 μV |
| Sahin. et al. ^[2]^ | Spiral cuff: platinum-foil and silastic | Hypoglossal nerve; cat | Anaesthetized | Recording, natural evoked | Vpp = 15 μV approximately, SNR = 2.24 ± 0.18 dB |
| Caravaca. et al. ^[3]^ | Spiral cuff: tungsten-titanium and epoxy | Vagus nerve; mice | Anaesthetized | Recording, pharmacological stimulation | Vpp = 100-2500 μV |
| Sahyouni. et al. ^[4]^ | Spiral cuff: platinum and silicon | Facial nerve; cat | Anaesthetized | Stimulation, multichannel | Selective facial muscle activation |
| Taylor and Durand ^[5]^ | Flat interface neural electrode (FINE): platinum foil and silicon | Sciatic nerve; cat | Anaesthetized | Stimulation, multichannel | Selective joint torque |
| Xiang. et al. ^[6]^ | Helical electrode: CNTs, Au, and polyimide | Sciatic nerve and peroneal nerve; rat | Anaesthetized | Recording, stimulation evoked, multichannel | Vpp = 125 μV |
| Chen. et al. ^[7]^ | Soft cuff: PEDOT-CNT and polyimide | Sciatic nerve; rat | Anaesthetized | Recording, natural evoked | Vpp = 150 μV |
| Lee. et al. ^[8]^ | Neural clip: polyamide-Au-polyimide and silicon | Vagus, sciatic and pelvic nerve; rat | Anaesthetized | Stimulation, multichannel | Control heart rate, muscle activation, voiding and baroreflex activation |
| Dong. et al. ^[9]^ | Multielectrode soft cuffs (MSC): PPy (DBS) and Au-coated perylene C (PaC) | Sciatic nerve; rat | Anaesthetized | Recording, natural evoked | Vpp = 11.38-29.42 μV |
| Carnicer-Lombarte. et al.^[10]^ | MSC: PEDOT:PSS and Au-coated PaC | Median, ulnar and radial nerves; rat | Wakefulness | Recording, spontaneous; stimulation, multichannel | Vpp = 14.6 ± 6.6 μV, SNR = 9.7 ± 6.1dB |
| Timothy M. et al. ^[11]^ | MSC: Au and polyimide, neural clip | Tracheosyringeal nerve; zebra finch | Wakefulness | Recording, spontaneous; stimulation, multichannel | Vpp = 3000 μV, SNR = 48.1 dB |
| Present work | Soft cuff: PEDOT:PSS and styrene-block-butadiene-block-styrene (SBS) | Facial nerve; rabbit | Wakefulness | Recording, spontaneous | Vpp = 84.00 ± 3.80 μV, SNR = 7.05 ± 1.78 dB |
| Spontaneous: activity arising from spontaneous behaviors or processes. Stimulation evoked: activity elicited by electrical stimulation of the implanted nerve. Natural evoked: activity elicited by naturalistic activation of sensory responses. | | | | | |

| **Table S2. Comparison of decoding models of peripheral nerve signals.** | | | | | |  |
| --- | --- | --- | --- | --- | --- | --- |
| Investigators | Experiment setting | Feature extraction | Decoding | Cross-subject decoding | Results |  |
| Nguyen. et al. ^[12]^ | Median and ulnar nerves, human; Longitudinal intra-fascicular electrodes (LIFE); Spike | Power spectrum | Convolutional neural network (CNN) + Long Short-Term Memory (LSTM) | No | Hand-movement intentions, variance accounted for (VAF) = 0.73 |  |
| Warren. et al. ^[13]^ | Median and ulnar nerves, human; Utah Slanted Electrode Array (USEA); Spike | Principal component analysis (PCA) | Kalman Filter | - | Finger movement, correlation coefficient (CC) = 0.78 |  |
| Davis. et al. ^[14]^ | Median and ulnar nerves, human; USEA; Spike | Spike firing rate | Kalman Filter | - | Finger movement, CC = 0.5 - 0.75 |  |
| Micera. et al. ^[15]^ | Median and ulnar nerves, human; LIFE; Spike | Classified spikes | Support vector machine (SVM) | No | Hand gesture, 4-class classification rate 0.85 |  |
| Cracchiolo. et al. ^[16]^ | Median and ulnar nerves, human; Transversal intrafascicular multichannel electrodes (TIMEs); Spike | PCA | SVM | No | Hand gesture, 11-class classification rate 0.83 |  |
| Eggers. et al. ^[17]^ | Sciatic nerve, canine; Flat interface nerve electrode (FINE); Population activity | None | Hybrid Bayesian Signal Extraction (HBSE) | No | EMG of the lower limb, CC = 0.82 ± 0.07, 0.61 ± 0.12 |  |
| Wang. et al. ^[18]^ | Sciatic nerve, rats; Spiked ultraflexible neural (SUN) interface; Spike | Threshold | SVM | - | Actual stimulation position of toes, accuracy = 44.4% - 77.8% |  |
| Present work | Facial nerve, rabbit; Soft cuff; Population activity | Handcrafted temporal feature / CNN + LSTM | Self-attention | Adaptive Prediction Head | Whisker movement trajectories, concordance correlation coefficient (CCC) = 0.68 ± 0.07 |  |
| No: only one subject; -: more than one subject but across subject decoding not mentioned. | | | | | |  |
|  |  |  |  |  |  |  |

**Table S3. WhiskerNet parameters size.**

| Network | Layer Type | Kernel Size | Input Size | Output Size | Stride & Padding |
| --- | --- | --- | --- | --- | --- |
| Handcrafted feature extraction network | LSTM | - | 160 ×1 | 30 × 1 | - |
|  | Fc | - | 30 × 1 | 30 × 1 | - |
| CNN feature extraction network | Conv1D | 25 × 1@16 | 2000 × 1 | 396 × 1@16 | 5 × 5, 0 × 0 |
|  | AvgPool | 5 × 4 | 16 × 396 | 16 × 100 | 1 ×4, 2 × 2 |
|  | Conv2D | 5 × 5 | 16 × 100 | 16 × 100 | 1 × 1, 2 × 2 |
|  | AvgPool | 5 × 3 | 16 × 100 | 16 × 33 | 1 × 3, 2 × 0 |
|  | Conv2D | 4 × 7 | 16 × 33 | 4 × 5 | 4 × 7, 0 × 1 |
|  | LSTM | - | 20 × 1 | 30 × 1 | - |
|  | Fc | - | 30 × 1 | 30 × 1 | - |
| Decoding part | Fc | - | 60× 1 | 60 × 1 | - |
|  | Self-Attention | 60 × 30 | 60 × 1 | 30 × 1 | - |
|  | Fc | - | 30 × 1 | 1 × 1 | - |

**Table S4. List of the handcrafted features.**

|  | Features | Formula |
| --- | --- | --- |
| 1 | Mean absolute value (MAV) | $\frac{1}{N}\sum_{i=1}^{N}\vert s\left[ i \right]\vert$ |
| 2 | Simple square integral (SSI) | $\frac{1}{N}\sum_{i=1}^{N}s\left[ i \right]^{2}$ |
| 3 | Standard deviation (STD) | $\sqrt{\frac{1}{N}\sum_{i=1}^{N}\left\vert s\left[ i \right]-\bar{s} \right\vert^{2}}$ |
| 4 | Average amplitude change (AAC) | $\frac{1}{N-1}\sum_{i=2}^{N}\vert s\left[ i \right]-s[i-1]\vert$ |
| 5 | Zero crossing (ZC) | $\sum_{i=2}^{N} \text{sgn}(-s[i-1]s[i])$ |
| 6 | Slope sign changes (SSC) | $\sum_{i=3}^{N} \text{sgn}(-(s\left[ i \right]-s[i-1])(s\left[ i-1 \right]-s[i-2]))$ |
| 7 | Maximum fractal length (MFL) | $\text{log}\sqrt{\sum_{i=2}^{N}\left\vert s\left[ i \right]-s[i-1] \right\vert^{2}}$ |
| 8 | Myopulse percentage rate (MPR) | $\sum_{i=1}^{N} \text{sgn}(\vert s\left[ i \right]\vert-s_{STD})$ |
| 9 | Difference absolute standard deviation (DABS) | $\sqrt{\frac{1}{N-1}\sum_{i=2}^{N}\left\vert s\left[ i \right]-s[i-1] \right\vert^{2}}$ |
| 10 | Log detector (LD) | $e^{\frac{1}{N}\sum_{i=1}^{N}\text{log}\vert s\left[ i \right]\vert}$ |

**Reference:**

[1] G. E. Loeb, R. A. Peck, *J. Neurosci. Methods* **1996**, *64*, 95

[2] M. Sahin, M. A. Haxhiu, D. M. Durand, I. A. Dreshaj, *J. Appl. Physiol.* **1997**, *83*, 317

[3] A. S. Caravaca, T. Tsaava, L. Goldman, H. Silverman, G. Riggott, S. S. Chavan, C. Bouton, K. J. Tracey, R. Desimone, E. S. Boyden, H. S. Sohal, P. S. Olofsson, *J. Neural Eng.*

**2017**, *14*, 066005

[4] R. Sahyouni, K. Goshtasbi, A. Presacco, J. Birkenbeuel, D. Cheung, A. Abiri, M. H. Berger, H. R. Djalilian, H. W. Lin, *Ann. Otol., Rhinol., Laryngol.* **2022**, *131*, 365

[5] M. D. Tarler, J. T. Mortimer, *IEEE Trans. Neural Syst. Rehabil. Eng.* **2003**, *11*, 227

[6] Z. Xiang, S. C. Yen, S. Sheshadri, J. Wang, S. Lee, Y. H. Liu, L. D. Liao, N. V. Thakor, C. Lee, *Adv. Mater.* **2016**, *28*, 4472

[7] N. Chen, B. Luo, A. C. Patil, J. Wang, G. G. L. Gammad, Z. Yi, X. Liu, S. C. Yen, S. Ramakrishna, N. V. Thakor, *ACS Nano* **2020**, *14*, 8059

[8] S. Lee, W. Y. X. Peh, J. Wang, F. Yang, J. S. Ho, N. V. Thakor, S. C. Yen, C. Lee, *Adv. Sci. (Weinh)* **2017**, *4*, 1700149

[9] C. Dong, A. Carnicer-Lombarte, F. Bonafe, B. Huang, S. Middya, A. Jin, X. Tao, S. Han, M. Bance, D. G. Barone, B. Fraboni, G. G. Malliaras, *Nat. Mater.* **2024**, *23*, 969

[10] A. Carnicer-Lombarte, A. J. Boys, A. Guemes, J. Gurke, S. Velasco-Bosom, S. Hilton, D. G. Barone, G. G. Malliaras, *Nat. Commun.* **2024**, *15*, 7523

[11] T. M. Otchy, C. Michas, B. Lee, K. Gopalan, V. Nerurkar, J. Gleick, D. Semu, L. Darkwa, B. J. Holinski, D. J. Chew, A. E. White, T. J. Gardner, *Nat. Commun.* **2020**, *11*, 4191

[12] A. T. Nguyen, J. Xu, M. Jiang, D. K. Luu, T. Wu, W.-k. Tam, W. Zhao, M. W. Drealan, C. K. Overstreet, Q. Zhao, *J. Neural Eng.* **2020**, *17*, 066001

[13] a) D. J. Warren, S. Kellis, J. G. Nieveen, S. M. Wendelken, H. Dantas, T. S. Davis, D. T. Hutchinson, R. A. Normann, G. A. Clark, V. J. Mathews, *Proc. IEEE* **2016**, *104*, 374; b) A. T. Nguyen, J. Xu, M. Jiang, D. K. Luu, T. Wu, W. K. Tam, W. Zhao, M. W. Drealan, C. K. Overstreet, Q. Zhao, J. Cheng, E. W. Keefer, Z. Yang, *J. Neural Eng.* **2020**, *17*,

[14] T. S. Davis, H. A. Wark, D. T. Hutchinson, D. J. Warren, K. O'Neill, T. Scheinblum, G. A. Clark, R. A. Normann, B. Greger, *J. Neural Eng.* **2016**, *13*, 036001

[15] S. Micera, P. M. Rossini, J. Rigosa, L. Citi, J. Carpaneto, S. Raspopovic, M. Tombini, C. Cipriani, G. Assenza, M. C. Carrozza, *J. NeuroEng. Rehabil.* **2011**, *8*, 1

[16] M. Cracchiolo, G. Valle, F. Petrini, I. Strauss, G. Granata, T. Stieglitz, P. M. Rossini, S. Raspopovic, A. Mazzoni, S. Micera, *J. Neural Eng.* **2020**, *17*, 026034

[17] a) T. E. Eggers, Y. M. Dweiri, G. A. McCallum, D. M. Durand, *J. Neural Eng.* **2017**, *14*, 056009; b) T. E. Eggers, Y. M. Dweiri, G. A. McCallum, D. M. Durand, *Sci. Rep.* **2018**, *8*, 14149

[18] J. Wang, X. Y. Thow, H. Wang, S. Lee, K. Voges, N. V. Thakor, S. C. Yen, C. Lee, *Adv. Healthcare Mater.* **2018**, *7*, 1700987
